# Clinical insights into tooth extraction via torsion method: a biomechanical analysis of the tooth-periodontal ligament complex

**DOI:** 10.3389/fbioe.2024.1479751

**Published:** 2024-10-10

**Authors:** Jiawei Xing, Guangzeng Zhang, Mingliang Sun, Hao Pan, Congdi Zhang, Yao Liu, Kehan Li, Ze He, Kailiang Zhang, Jizeng Wang, En Luo, Baoping Zhang

**Affiliations:** ^1^ Department (Hospital) of Stomatology, Lanzhou University, Lanzhou, China; ^2^ State Key Laboratory of Oral Diseases & National Center for Stomatology & National Clinical Research Center for Oral Diseases, West China Hospital of Stomatology, Sichuan University, Chengdu, Sichuan, China; ^3^ Key Laboratory of Mechanics on Disaster and Environment in Western China, Ministry of Education, College of Civil Engineering and Mechanics, Lanzhou University, Lanzhou, China; ^4^ Key Lab of Maxillofacial Reconstruction and Intelligent Manufacturing, Lanzhou, Gansu, China

**Keywords:** biomechanics, periodontal ligament, minimally invasive dentistry, finite element analysis, oral surgery

## Abstract

Traditionally, extracting single, flat- or curved-rooted teeth through twisting is unfeasible. However, our clinical practice suggests that such teeth can be extracted efficiently through moderate twisting in a minimally invasive manner. Given the lack of studies on biomechanics of the tooth–periodontal ligament (PDL) complex during torsion, which has further constrained its application, we assessed the feasibility of the torsion method for extracting single-rooted teeth and evaluated its minimally invasive potential. Using three-dimensional finite element analysis, we examined the stress distribution of the tooth and PDL during torsion. Then, we examined changes in the optimal torsion angle (OTA) and stress distribution across various anatomical scenarios. During torsion loading, stress concentration was primarily observed on the sing-rooted tooth surface near the alveolar crest, whereas molars at the root furcation. The OTA was found to increase under conditions such as narrowing of root width, decrease in the root apical curvature, change from type I to IV bone, alveolar bone loss, and shortening of root length. Moreover, the clinically validated model demonstrated that 74% of outcomes fell within the standard OTA range. In conclusion, the decrease in PDL area necessitated a larger angle for complete PDL tearing. Single-rooted teeth with root width-to-thickness ratios of ≥0.42 and apical curvatures of ≤30°are suitable for extraction using the torsion method. This study confirms the feasibility of the torsion method for minimally invasive tooth extraction and expands its indications, laying the theoretical foundation and essential insights for its clinical application.

## Graphical Abstract

**Figure:**
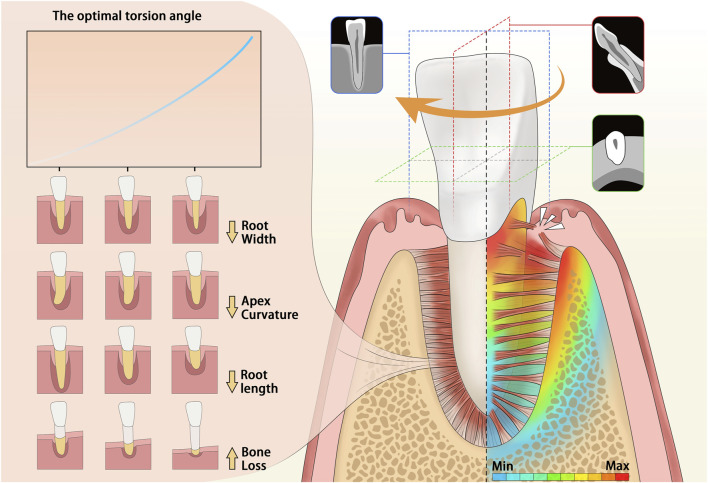
Graphical abstract: This is a graphical representation of the abstract. During torsion, the highest stress is concentrated at the cervical region of the single-rooted tooth. The impact of various anatomical conditions on the optimal torsion angle (OTA) is illustrated on the left side of the figure. As the root narrows, the apical curvature angle decreases, the Type of bone I to IV, the alveolar bone loss occurs, or the root shortens, and the required optimal torsion angle increases.

## Introduction

Various dental conditions, such as malocclusion and severe periodontitis, can cause irreversible damage to teeth, often necessitating their extraction (McCaul et al., 2001; Jain et al., 2024). Traditional extraction tools, such as bone chisels and bone hammers, often lead to severe complications including tissue inflammation, (Daly et al., 2022) nerve injury, (Khavanin et al., 2019; Bailey et al., 2020), and even alveolar bone fractures (Zhao et al., 2023). Since Cyrus Fay’s pioneering invention of extraction forceps in 1827, (Moskow, 1987) they represent the most fundamental and extensively used instruments. With rapid advancements in precision medicine and minimally invasive principles, minimally invasive extraction techniques have gained high popularity (Kelly et al., 2016; Spinato et al., 2016; Nehme et al., 2021). In this context, minimally invasive tooth extraction has increased the demand for forceps that are minimally invasive. Extraction of the affected tooth by using dental forceps involves twisting, swinging, and applying traction, which can cause damage to the alveolar bone and even to the surrounding teeth or periodontal tissues with improper use (Bailey et al., 2020; Zhao et al., 2023). However, the variability in tooth and periodontal anatomy, combined with a lack of quantitative research on tooth extraction biomechanics, has hindered progress in achieving consistently successful outcomes.

The periodontal ligament (PDL) and alveolar bone are pivotal in supporting, transmitting, and distributing forces within the mouth (Beertsen et al., 1997; Lin et al., 2020). Efficiently severing the PDL and minimizing damage to the alveolar bone are crucial for successful tooth extraction. Conventional wisdom suggests that teeth with a single, round root, such as the maxillary central incisor and canine, can be extracted using torsional forces. However, during the extraction of teeth with flat, curved, or multiple roots, improper application of torsional force often leads to root fractures and other complications (Cicciù et al., 2013). Torsional loading, a technique involving repeated rotation along the longitudinal axis of the tooth root, (Moga et al., 2022) generates substantial shear forces on the PDL. According to our clinical experience with dental forceps, teeth with single, flat or curved roots can be moderately twisted during extraction without substantial complications, such as root fractures. We hypothesize that applying torsion for the extraction of single-rooted teeth can make the process efficient and less traumatic. Based on biomechanical analyses of the tooth–PDL bone complex are instrumental in addressing clinical problems in dentistry.

This study investigated the biomechanical behavior of the tooth-PDL complex during torsion and examined the applicability of employing torsion extraction under diverse anatomical conditions. We performed three-dimensional finite element analysis to determine the distribution of stress within the tooth-PDL complex during torsion, and the effect of torsion speed on the optimal torsion angle (OTA). Furthermore, we determined the OTA and its variation pattern under five anatomical models (root width, root apex curvature, Type I to IV bone, alveolar bone loss and root length groups) based on the PDL failure criteria and observed the differences in stress distribution to clarify the feasibility of the torsion method. The reliability of our results is further validated through clinical case data. The findings of this study can enhance our understanding regarding the biomechanical foundation of torsion extraction in clinical practice and provide a novel strategy to advancing minimally invasive extraction technology and refining extraction instruments.

## Materials and methods

**FIGURE 1:**
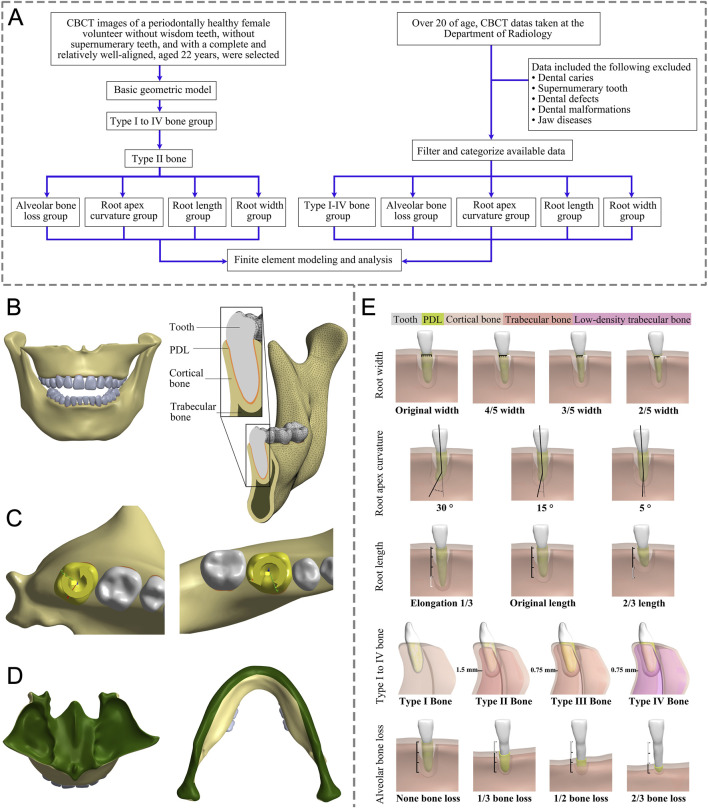
**(A)** This flow chart outlines the key steps in our study. **(B)** Front view and detail of a 3D FE model. **(C)** Load conditions. **(D)** Boundary conditions (green region). **(E)** Modeling based on different anatomical structures: Root width group (from left to right: original width, 4/5, 3/5, and 2/5 width of original); Root length group (from left to right: Elongation by 1/3, original length, 2/3 length of the original; Root apex curvature group (from left to right, apical curvatures of 30°, 15°, and 5°); Type I to IV bone group (from left to right: Type I to IV bone); Alveolar bone loss group (from left to right: bone loss as none, 1/3, 1/2, and 2/3 bone loss); FE, finite element; PDL, periodontal ligament.

The study protocol was approved by the Ethics Committee of the School of Stomatology, Lanzhou University (No. LZUKQ-2024-046) and was performed in accordance with the guidelines of the Declaration of Helsinki (2013 revision). The written informed consent was obtained from subjects for each CBCT data. The study design is illustrated in Figure 1A and element and node statistics are shown in Supplementary Tables S1, S2.

### Basic geometry modeling

CBCT (KAVO, Germany, 120 kVp, 5 mA, 0.3 mm) images data from a 22-year-old female with healthy periodontal tissue, no impacted opsigenes, no extra teeth, and an intact and regular dentition were selected. Mimics (21.0, Materialise, Belgium) and Geomagic Wrap (2021, 3D Systems, SC, United States) were utilized to create individual three-dimensional solid models for the maxilla, mandible, and all 28 teeth, respectively. SolidWorks (2017, Dassault Systemes, France) was employed to create PDL models with a thickness of 0.25 mm (Qian et al., 2009; Begum et al., 2015; Cattaneo and Cornelis, 2021). The parts of the teeth, PDL, maxilla, and mandible were assembled separately without interference, and a geometric model was obtained (Figure 1B). Then Ansys Workbench (2019R2, Ansys, PA, United States) was imported to set the teeth-PDL and PDL-alveolar bone as a bonded contact relationship and assigned the material properties through parameters (Table 1). (Chatvanitkul and Lertchirakarn, 2010; Tanne et al., 1991; Morgan et al., 2018; Tribst et al., 2024; Geramy, 2000; Tsouknidas et al., 2016; Toms et al., 2002; Rudolph et al., 2001) Given the irregular shape of the model, the mesh was primarily composed of hexahedrons, supplemented by tetrahedrons. After mesh independent study, the mesh was divided into 0.7 mm size. The sphere that shared the center with the tooth axis further refined the grid of alveolar bone around the teeth. Based on the aforementioned foundational model construction process, built the following grouped models.

**TABLE 1:** Materials properties of the finite element model.

Material	Material model	Coefficients	Reference
Cortical bone	Elastic	E = 13,700 MPa, ν = 0.26 ρ = 1,400 kg/m^3^	Tanne et al. (1991); Chatvanitkul and Lertchirakarn (2010); Morgan et al. (2018)
Trabecular bone	Elastic	E = 1,370 MPa, ν = 0.3 ρ = 1,400 kg/m^3^	Geramy (2000); Chatvanitkul and Lertchirakarn (2010); Tsouknidas et al. (2016); Tribst et al. (2024)
Low-density trabecular bone	Elastic	E = 231 MPa, ν = 0.3 ρ = 1,400 kg/m^3^	Tsouknidas et al. (2016)
Tooth	Elastic	E = 19,600 MPa, ν = 0.30 ρ = 2,200 kg/m^3^	Tanne et al. (1991); Chatvanitkul and Lertchirakarn (2010)
Periodontal ligament	Elastic	E = 0.667 MPa, ν = 0.45 ρ = 1,100 kg/m^3^	Tanne et al. (1991); Rudolph et al. (2001); Toms et al. (2002)
	Viscoelastic	G_1_ = 0.0897, G_2_ = 0.1093 G_3_ = 0.7852 MPa $\tau_{1}$ = 0.1548, $\tau_{2}$ = 0.0038 $\tau_{3}$ = 3.521 × $10^{-5}$	Chatvanitkul and Lertchirakarn (2010); Toms et al. (2002)

Note: *E*: elastic modulus; *ν*: Poisson’s ratio; *ρ*: density.

$G\left(t\right)=G_{1}e^{-\tau_{1}t}+G_{2}e^{-\tau_{2}t}+G_{3}e^{-\tau_{3}t}$ , (G: reduced relaxation function; τ: decay constant).

### Modeling of the type I to IV bone group

Based on the differences in the thickness and components of cortical and trabecular bone (Supplementary Table S3), the jaw is categorized into four bone types according to previous studies: Type I bone primarily consists of cortical bone; Type II bone comprises a 1.5 mm thick external layer of cortical bone and an internal layer of trabecular bone; Type III bone features an outer layer of cortical bone with an inner layer of 0.75-mm-thick trabecular bone; and Type IV bone is composed of a 0.75 mm thick outer layer of cortical bone and internal low-density trabecular bone (Morgan et al., 2018; Tribst et al., 2024). The initial step involved offsetting the processed jaw bone model as a whole using Geomagic Wrap, with the thickness determined by the bone type. This process yielded a preliminary trabecular bone model. Following this, both the original jaw bone model and the trabecular bone model were imported into SolidWorks to generate the cortical bone model. The obtained models of cortical bone, trabecular bone, teeth, and PDL were assembled and examined to confirm that there was no interference between the models, and the Type I to IV bone group models were obtained (Figure 1E). Studies have demonstrated that human jaws are predominantly composed of type II bone (53.33%), followed by Type III bone (26.67%) (Geramy, 2000). Following the principle of controlling variables, we subdivided groups of root width, root apex curvature, alveolar bone loss, and root length, based on Type II bone.

### Modeling of the root width group

The normal tooth models were imported into Geomagic Wrap, we measured the cervical root widths using the Distance tool in Geomagic Wrap and created models of different root widths after deleting the excess according to the desired width, resulting in tooth models with original widths of 2/5, 3/5, and 4/5, and the root cervical width-to-thickness ratio (mesial and distal diameter/buccolingual diameter) was measured. The rest of the procedure was the same as for the basic model (Figure 1E).

### Modeling of the root apex curvature group

Apex curvature is a common anatomical variation of teeth. The Unigraphics NX (10.0, Siemens PLM Software) was used to determine the position of the apical third of the root. The original angle of the root tip at this location was measured. Subsequently, the apical third of the root was selected as the moving object and the tip was bent in the distal direction to obtain apical curvature models of 5°, 15°, and 30°, respectively. The combined perfect models were then imported into Geomagic Wrap, where subsequent steps followed the procedures outlined in the basic model above (Figure 1E).

### Modeling of the alveolar bone loss group

Age-related physiological alveolar bone resorption occurs, (Baima et al., 2022), and factors such as smoking, periodontal disease, and systemic illnesses have been linked to pathological alveolar bone loss (Albandar, 1990; Yu X et al., 2022). To discern the impact of alveolar bone loss on OTA, we modeled teeth with no loss of alveolar bone and with bone losses of 1/3, 1/2, and 2/3 of the root length. The root length was measured using the Distance tool within Geomagic Wrap from the tooth cervix to the apex. The Deform Region tool was employed to define the corresponding alveolar crest area. Subsequently, the alveolar crest was reduced based on each root’s length to create the absorption model. The remaining steps were the same as the basic model (Figure 1E).

### Modeling of the root length group

In Geomagic Wrap, the root length was adjusted based on the root length measurement data, resulting in the creation of the root length variation model using the basic method mentioned above (Figure 1E).

### Construction of the theoretical clinical validation model

In this study, CBCT images were selected from patients over 20 years at the Department of Radiology, Lanzhou University Stomatology Hospital, Lanzhou, China, from June 14 to 30 June 2024, and a total of 31 teeth were enrolled. The inclusion criteria required subjects to possess a minimum of 28 teeth (excluding third molars) and for these teeth to be free from dental conditions such as caries, defects, and malformations. The data of root length, root width, root apex curvature, root perimeter six-point cortical bone thickness (Supplementary Figure S1), and alveolar bone height absorption for the teeth under study were obtained based on the CBCT. Three clinicians with 5 years of clinical experience grouped the teeth based on criteria including Type I to IV bone group, alveolar bone loss group, root apex curvature group, root length group, and root width group. Modeling was performed using the same methods as described above (Supplementary Table S4).

### Loading and boundary conditions

Due to the high symmetry of homonymous teeth in the same jaw, we selected right-side teeth for analysis. Although applying a force couple at the midpoint of the clinical crown on the buccal and lingual sides of the tooth for mechanical analysis was feasible, a uniform matching relationship between the magnitude of the force couple and the torsion angle could not be achieved. Therefore, using displacement loading was considered more appropriate and accurate. In this study, rotational loading of 15° (maxillary teeth in counterclockwise, mandibular teeth in clockwise) was set to be carried out independently along the long axis of the teeth (Figure 1C). The sub-steps settings were set ensuring that the change in time and the torsion angle corresponded linearly in the analysis process, that is, the torsion process was a uniform motion so that the stress value of the corresponding angle at any time in the torsion process could be obtained.

In this process, the upper area of the maxillary model was fixed, and the mandible model was fixed from the surface of the condyle on one side along the ascending mandibular branch, the inferior border of the mandible, and the ascending mandibular branch of the contralateral side to the surface of the contralateral condyle to ensure that the jaws were stationary during tooth torsion (Figure 1D).

### Failure criterion

We assumed that the PDL would be completely torn when the minimum von Mises stress in the PDL model exceeded the established threshold of 0.026 MPa. (Tribst et al., 2024; Lee, 1965; Lee et al., 2018; Youssef et al., 2020). Furthermore, we monitored σ_max_ and σ_min_ in the tooth root area to ensure they did not exceed the ultimate tensile strength (52.9 MPa) and ultimate compressive strength (260 MPa), respectively. (Morgan et al., 2018; Lee, 1965; Watts et al., 1987; Hou and Tsai, 1997). The specific angle at which these conditions were met—representing the minimum angle of unidirectional rotation around the tooth’s long axis necessary to fully tear the PDL—was identified as the OTA. This angle satisfies the established failure criteria for tooth extraction.

## Results

### Stress concentrates near the alveolar crest on the tooth surface (molars at root furcation)

**FIGURE 2:**
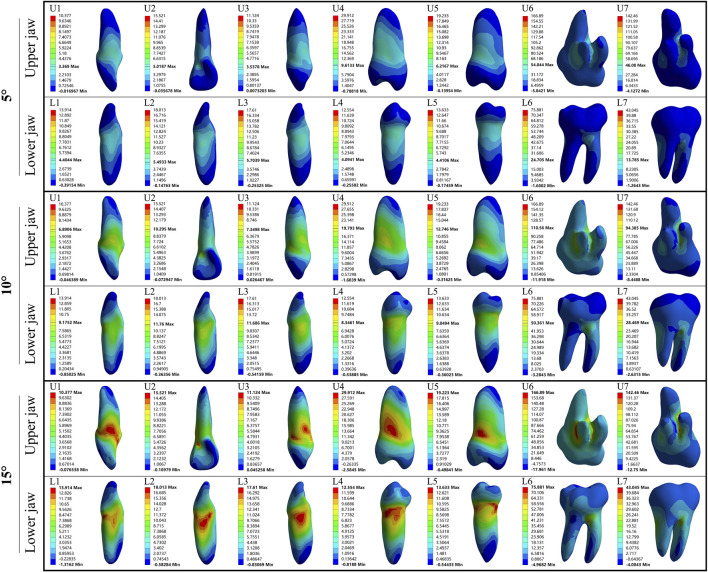
The maximum principal stress distribution of the tooth during 5°–15° torsion. U: upper jaw; L: Lower jaw.

**FIGURE 3:**
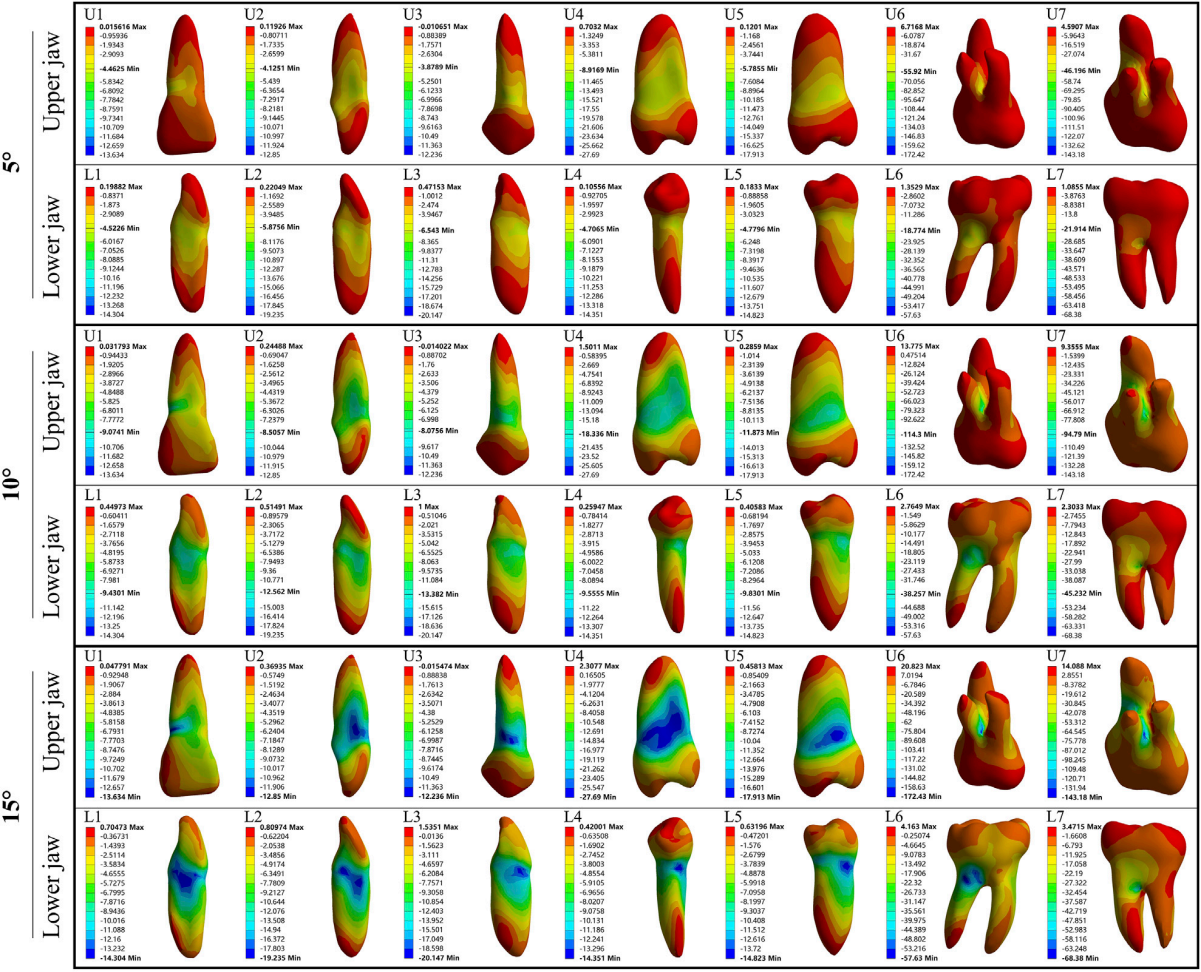
The minimum principal stress distribution of the tooth during 5°–15° torsion. U: upper jaw; L: Lower jaw.

In the upper jaw (U1, U2, and U3), stress was the most concentrated on the labial tooth cervix. However, in the lower jaw (L1, L2, and L3), this stress was predominantly concentrated on the mesial-distal tooth cervix. In upper premolars (U4 and U5), the stress was concentrated on the mesial-distal tooth cervix. A similar pattern was observed in lower premolars (L4 and L5), with stress being concentrated at the mesial-distal tooth cervix. The stress of U6, U7, L6, and L7 was concentrated at the root furcation. The minimum stress point for all the aforementioned teeth was located at the root apex (Figures 2, 3). Except for U6 and U7 where the PDL exhibited stress concentration at the palatal root apex, the remaining PDL behaved similarly to their respective teeth (Supplementary Figure S2).

### Narrow roots require a large OTA

**FIGURE 4:**
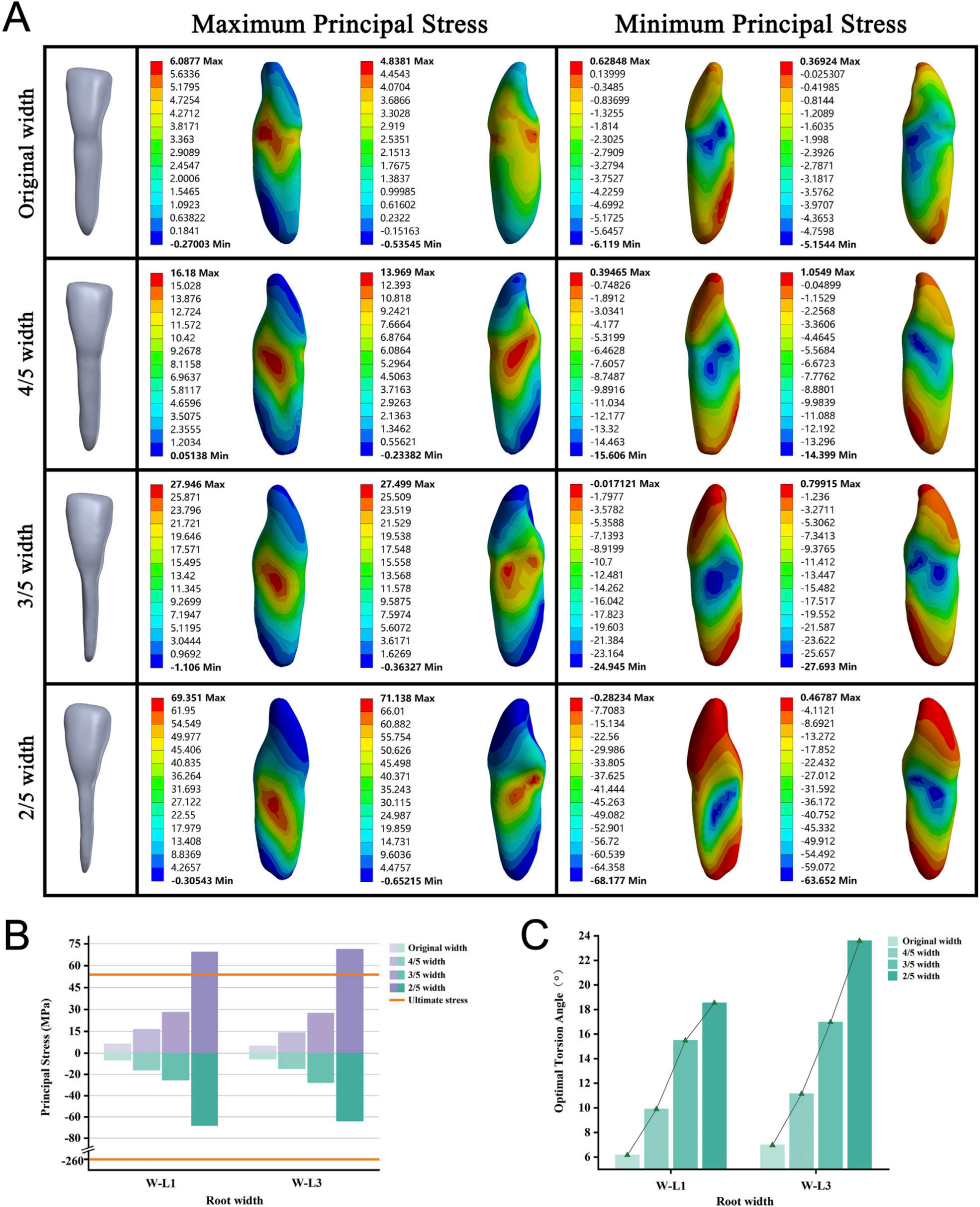
**(A)** Principal stress distribution in teeth of root width group at the optimal torsion angle. **(B)** Principal stresses on root width groups. **(C)** The optimal torsion angle of root width groups. W–L: Root Width group-Lower jaw.

We focused on single, flat-rooted teeth to construct and analyze tooth models with original root widths reduced to 4/5, 3/5, and 2/5 of their full size. As the root width decreased, all models required a larger OTA to achieve complete PDL rupture (Figure 4C). No root fractures occurred when the root width was reduced to 3/5 of its original size. However, reducing the root width to 2/5 of the original size resulted in fractures in all roots before the incidence of complete PDL rupture (Figure 4B). With the narrowing of the root, stress concentration on the teeth gradually increased, shifting from the tooth surface near the alveolar crest to the middle of the root, whereas the apical region continued to experience the least stress (Figure 4A; Supplementary Figure S3).

### Large root apex curvature necessitates a small OTA

**FIGURE 5:**
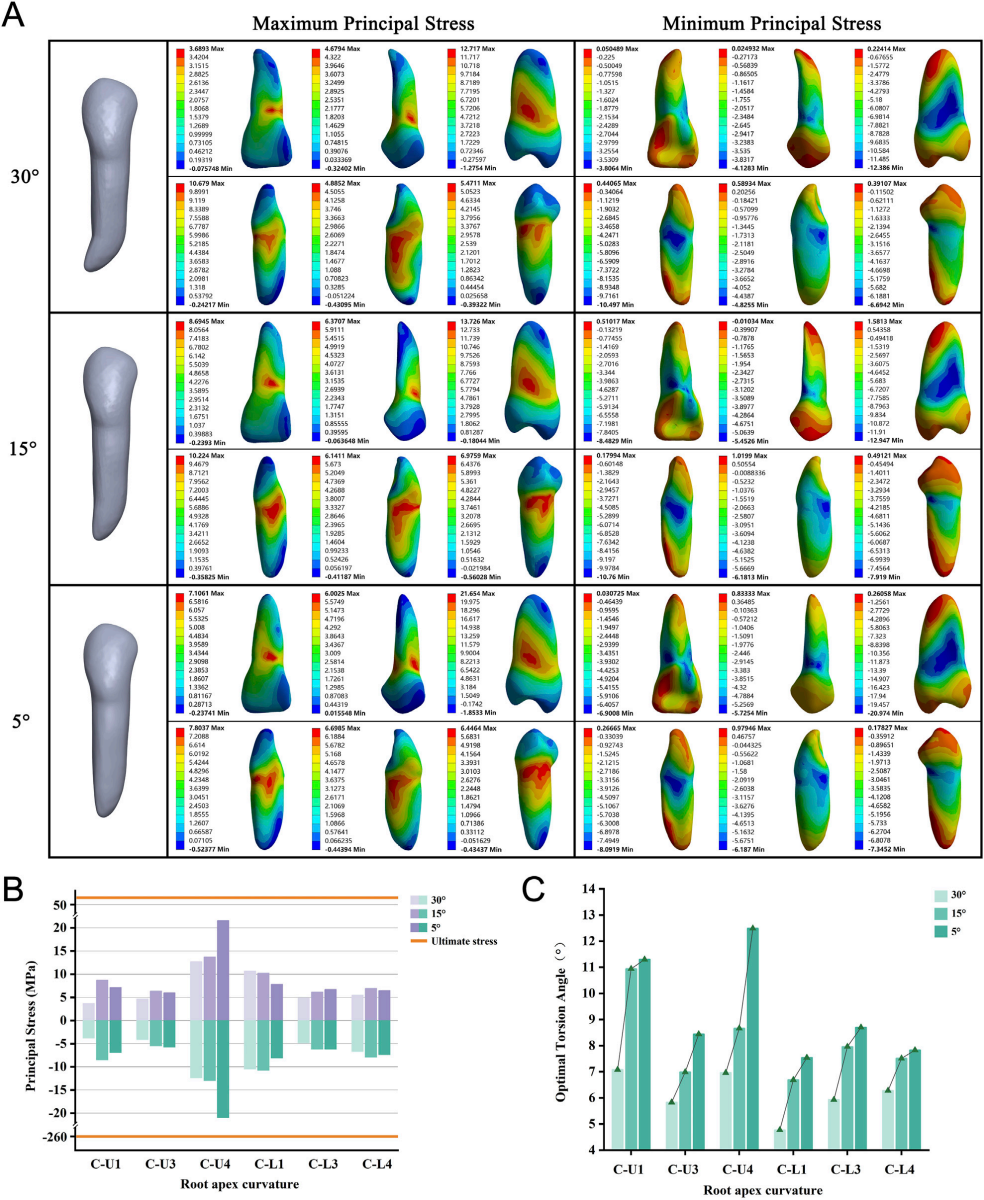
**(A)** Principal stress distribution in teeth of root apex curvature group at the optimal torsion angle. **(B)** Principal stresses on root apex curvature group. **(C)** The optimal torsion angle of root apex curvature group. C-U/L: Root Apex Curvature group-Upper/Lower jaw.

We included six teeth, each modeled with apical curvatures of 5°, 15°, and 30°. We observed that as the apical curvature increased, the required OTA tended to decrease for all models (Figure 5C). None of the roots experienced fractures when the PDL was completely ruptured (Figure 5B). During torsion, the maximum stress was consistently located at the alveolar crest, whereas the minimum stress was centered on the root shifted from the apex to the curved convex surface (Figure 5A; Supplementary Figure S4).

### OTA increases from type I to IV bone

**FIGURE 6:**
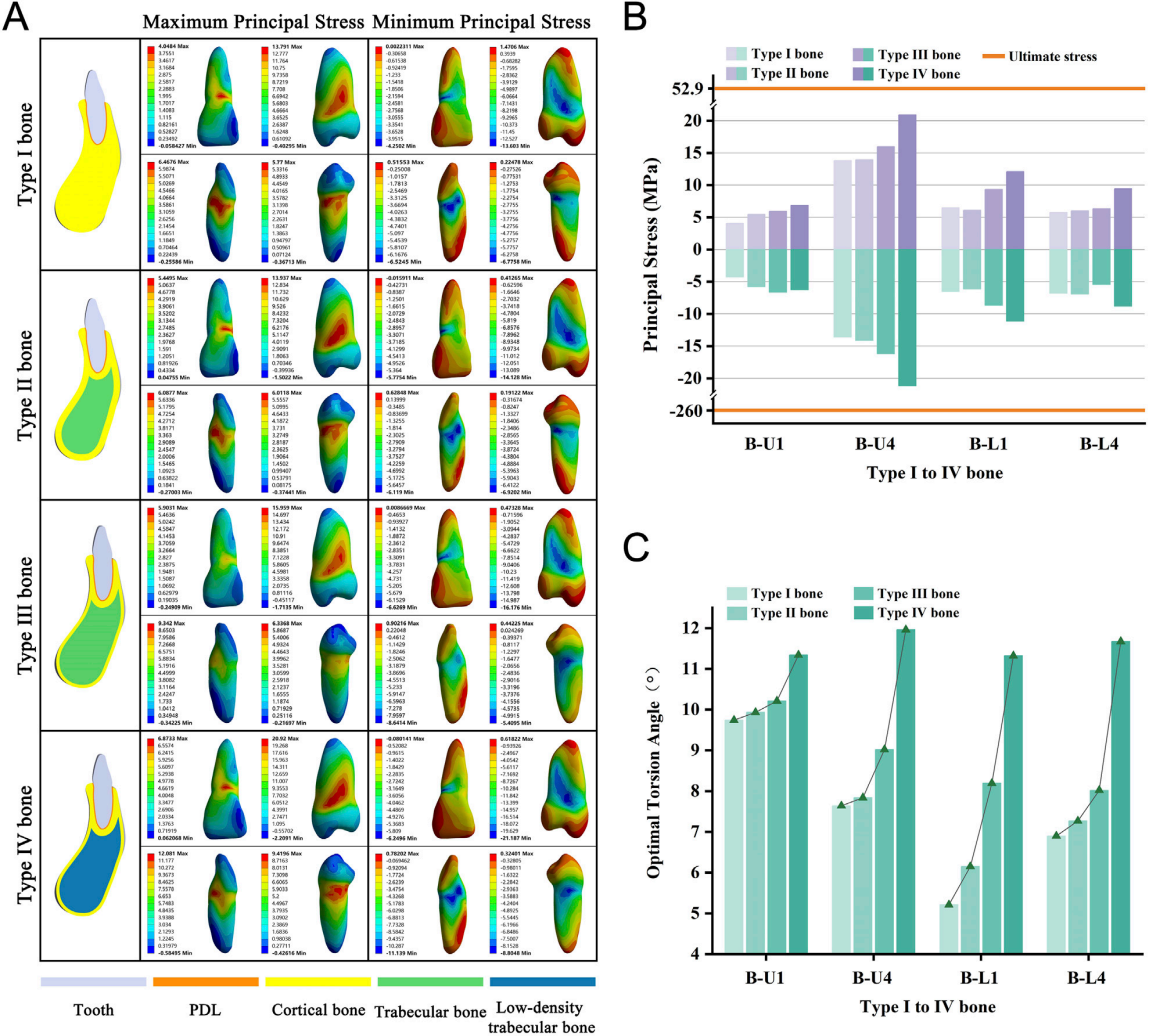
**(A)** Principal stress distribution in teeth of Type I to IV bone group at the optimal torsion angle. **(B)** Principal stresses on Type I to IV bone group. **(C)** The optimal torsion angle of Type I to IV bone group. B-U/L: Type I to IV Bone group-Upper/Lower jaw.

We selected four teeth from the anterior and premolar regions of both the upper and lower jaws, representing bone Types I through IV. These types indicate a progression from denser to less dense bone, characterized by decreasing cortical bone thickness and trabecular bone density. As we transitioned from Type I to Type IV bone, we observed a consistent increase in the required OTA across all models (Figure 6C). None of the teeth experienced fractures when the PDL was completely torn (Figure 6B). The stress distribution observed aligned with the predictions of the basic model (Figure 6A; Supplementary Figure S5).

### Alveolar bone loss increases the required OTA

**FIGURE 7:**
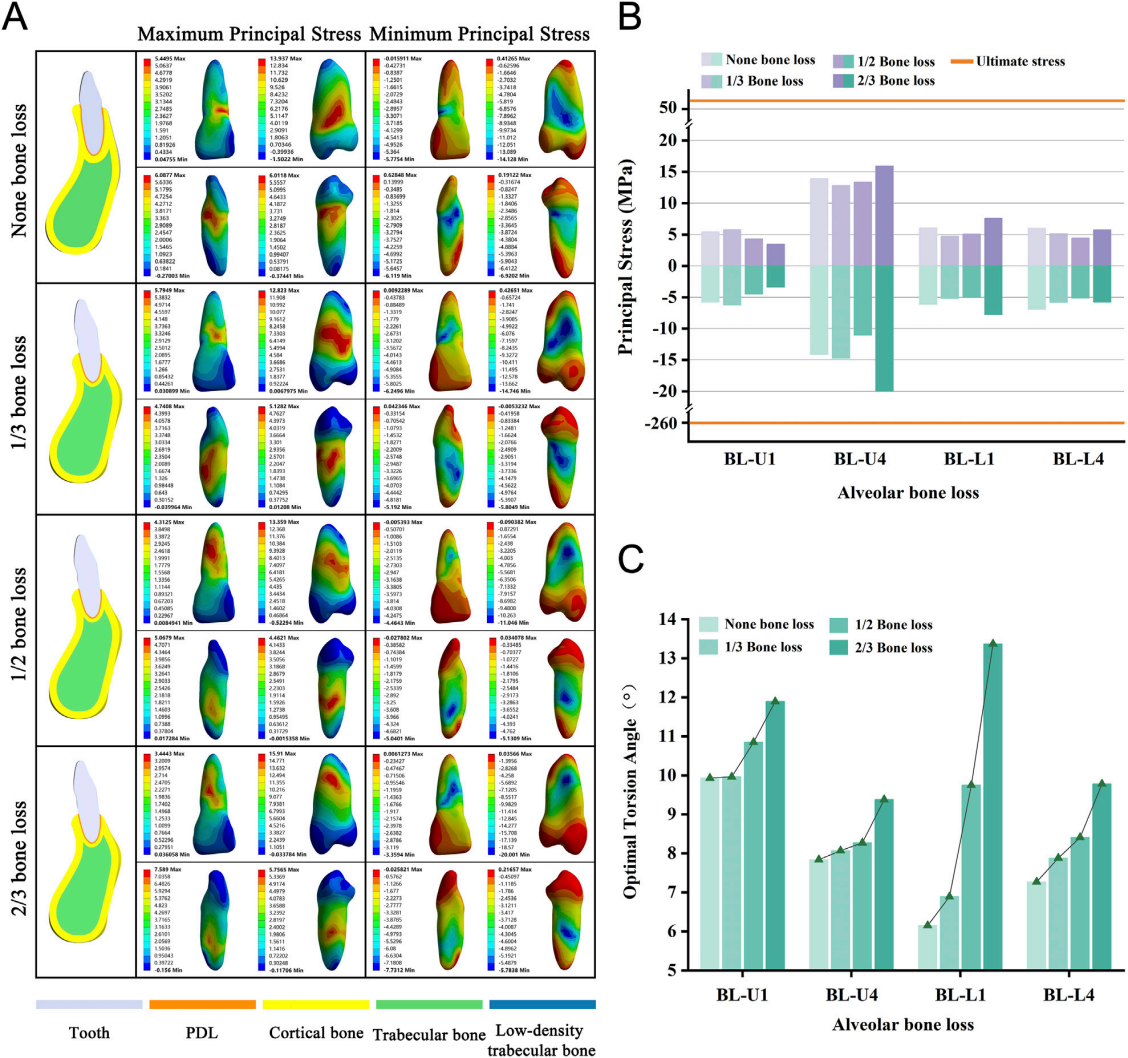
**(A)** Principal stress distribution in teeth of bone loss group at the optimal torsion angle. **(B)** Principal stresses on bone loss group. **(C)** The optimal torsion angle of alveolar bone loss group. BL-U/L: Alveolar Bone Loss group-Upper/Lower jaw.

We selected four teeth and categorized them based on the extent of alveolar bone loss into four groups, namely none, 1/3, 1/2, and 2/3, for modeling purposes. As the degree of alveolar bone loss increased, the required OTA also showed a consistent upward trend across all models (Figure 7C). None of the teeth experienced fractures when the PDL was completely torn (Figure 7B). In addition, with increasing bone loss, stress concentration consistently occurred on the tooth surface near the alveolar crest (Figure 7A; Supplementary Figure S5).

### Long roots require a small OTA

**FIGURE 8:**
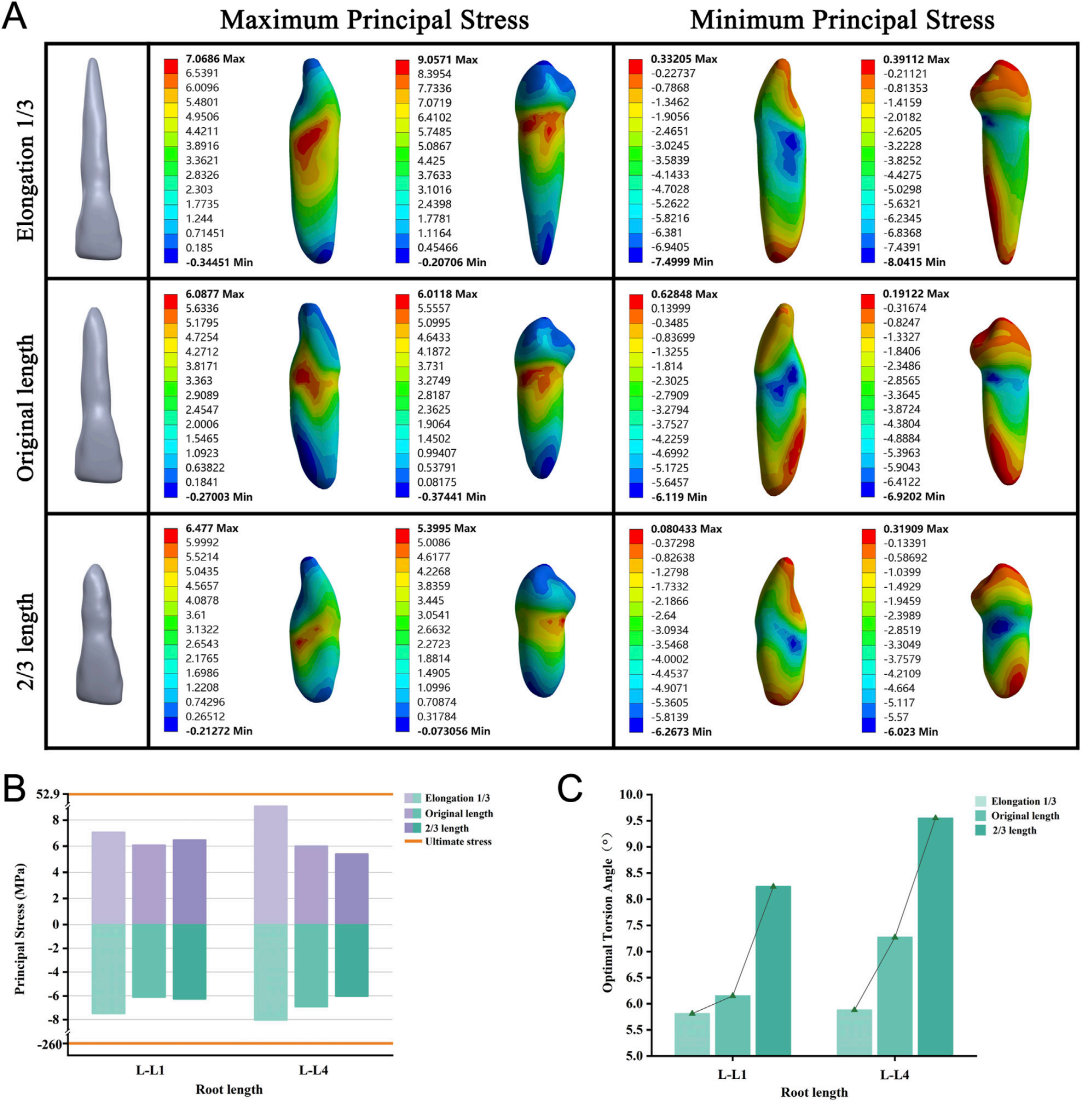
**(A)** Principal stress distribution in teeth of root length group at the optimal torsion angle. **(B)** Principal stresses on root length group. **(C)** The optimal torsion angle of root length group. L–L: Root Length group-Lower jaw.

To eliminate the effect of the maxillary sinus on our root elongation model, we simulated the roots of lengths 2/3 of the original, original length root, and elongation by an additional 1/3. We observed a decreasing trend in the OTA as the root length increased (Figure 8C). No tooth fractures occurred in any of the groups when the PDL was completely ruptured (Figure 8B). Throughout the variations in length, the stress concentration remained primarily on the tooth surface near the alveolar crest, whereas the minimum stress consistently appeared at the root apex (Figure 8A; Supplementary Figure S7).

### Theoretical analysis of clinical case data

**FIGURE 9:**
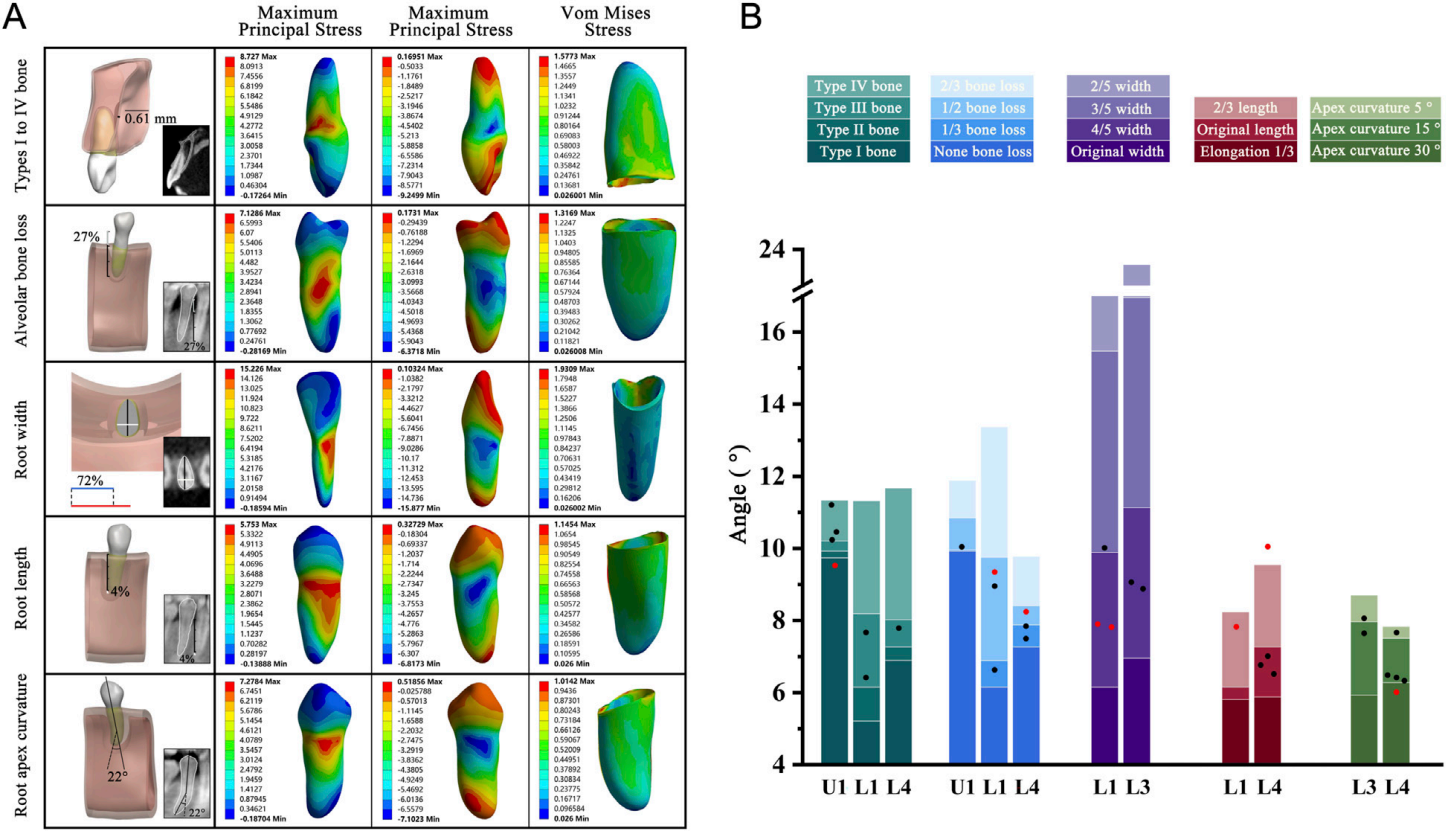
**(A)** Geometric model, CBCT images, and teeth principal stresses distribution. Type I to IV bone group: The average thickness of cortical bone in this case model was 0.61 mm; Alveolar bone loss: Alveolar bone height lost 27% of original height (white); Root width group: In the case model, the ratio of mesiodistal diameter (white) to buccolingual diameter (black) was represented in blue, while the basic model was represented in red. Blue to red indicated that the width-to-thickness ratio was reduced to 72% compared to the basic model; Root length group: The root length was 4% longer than the basic model (white); Root apex curvature group: The apical curved 22° toward the distal. **(B)** The five different anatomical model results were presented in the form of stacked bar charts for OTA ranges, with clinical case verification results presented in the graphical form of scatter points. Red dot Indicated that the case OTA exceeded the corresponding standard OTA range. OTA: the optimal torsion angle; U: Upper jaw; L: Lower jaw.

The PDL was successfully ruptured in all cases, none of which exhibited root fracture when subjected to torsion. Notably, 74% of these cases fell within the OTA range, aligning with the patterns observed under various anatomical conditions. This consistent pattern provides a clinical rationale for using this method. Furthermore, stress distribution observed in the teeth and PDL corresponded closely with the patterns described in our anatomical structure models (Figure 9; Supplementary Table S4).

## Discussion

In this study, we utilized finite element analysis to elucidate the stress distribution within the tooth-periodontal complex during the torsion process of tooth extraction. By different anatomical conditions of the tooth and periodontal structures, we identified change patterns and ranges of OTA, thereby confirming the safety and broad applicability of the torsion method.

### Force application in tooth extraction: impact on the stress state of the tooth-PDL-alveolar bone complex

In the context of tooth extraction, PDL tearing is critical in facilitating tooth dislocation (Qian et al., 2009). Traditional dental forceps utilize a combination of swinging, twisting, and traction forces to tear the PDL. These methods are governed by unique mechanical principles that yield varying outcomes when combined with minimally invasive extraction instruments (Coulthard et al., 2014). The swing force relies on the principle of alveolar bone flexibility, involving repeated compression and expansion of the alveolar socket, tearing the PDL in the direction opposite to that of the applied force and loosening the tooth for extraction (Ojala and Lehtinen, 1980). However, the swing method applies pressure to the buccal and lingual alveolar bone, it is easy to cause the height of the buccal and lingual alveolar bone wall to be destroyed. In cases involving the lingual side of the mandible or older patients with thinner bone plates due to resorption, significant deformation of the bone plate is more likely to cause alveolar bone fractures (Baniwal et al., 2007). By contrast, the twisting method involves inserting dental forceps into the tooth cervix and gently twisting them along the tooth’s long axis to effectively disrupt the horizontal group in the PDL (Menchini-Fabris et al., 2022). Torsion minimizes the excessive enlargement of the alveolar socket, thus reducing postoperative complications (Lehtinen and Ojala, 1980; Al-Khateeb and Alnahar, 2008). Moreover, our stress distribution analysis indicated that torsion led to a more uniform stress distribution across the root surface, effectively reducing the risk of inadvertent root fractures. Some studies applied torque to teeth during orthodontic treatment, and the tooth-periodontal ligament complex exhibited a stress distribution pattern similar to that observed in our study (Cheng Y et al., 2022; Zhu GY et al., 2023; Meng X et al., 2023). Given these advantages, the torsion method has wider application potential in single-rooted teeth. Further understanding of stress distribution in different anatomical structures, along with mastering the pattern of OTA, will define the scope of the torsion method’s application clearly.

### Impact of anatomical structure on extraction feasibility

In clinical and dental education, the torsion method has been limited to flat and curved roots (Hupp et al., 2018). We analyzed the performance of the torsion method on flat and curved roots with varying degrees of variability and found interesting results. Flat-rooted teeth have more pronounced root surface depressions on the mesial and distal surfaces, where stresses are mainly concentrated. As the root narrows, the stress concentration area gradually moves downward. However, flatter single-rooted teeth (L1, L3) in the full dentition fractured only at 2/5 of the original width (width-to-thickness ratios of 0.34). Flatter roots required a greater angle of twist to tear all the PDL, creating greater stresses in progressively weaker areas and leading to fractures. Notably, this extreme model is rare in clinical (Martins et al., 2020; Nelson, 2019). Based on these results, we concluded that single-rooted teeth with root width-to-thickness ratios of 0.42 (3/5 width) or more can be extracted using the twisting method. In studying curved-rooted teeth, we found that stress concentration was not at the curved part of the root but at the cervical part of the tooth. As the bending angle increased, stress in the apical region rose but remained much lower than in the cervical region. This indicates that the torsion method is still viable for teeth with apical bending angles of 30° or less. This optimizes the mechanical efficiency of the extraction and reduces the potential risk of root fracture.

In the analysis of alveolar bone type, bone loss, and root length groups, changes in periodontal tissue anatomy affect the OTA. From Type I to Type IV bone, cortical bone thickness and trabecular bone density decrease, and the loss of alveolar bone and shortening of the root reduce PDL area. These factors diminish binding forces in the alveolar socket, allowing less stress on the tooth-PDL-bone complex for the same torsional force, enabling a greater twisting angle.

### Theoretical analysis of clinical case data

In clinical settings, individual variations among patients directly affect the choice of the extraction method. These differences encompass the patient’s dental and periodontal conditions compared with theoretical simulations. Therefore, establishing clinically relevant case models and calculating the OTA are valuable for determining the applicability of the standard OTA range in diverse clinical scenarios. After CBCT-based modeling analysis of clinical cases, we found 74% of results within the standard OTA range, while 26% were not applicable. We considered that the reason for the deviation could be the concomitant alteration of the tooth by multiple influencing factors or unexplored anatomical variations. The OTA is determined based on a standard model. The anatomical morphology of each tooth is highly individualized. For example, the bending direction of the root apex may deviate, and root surfaces can have irregular depressions or protrusions. These factors can cause stress imbalances during torsional loading, leading to shifts in the optimal torsional angle. Moreover, in the clinical CBCT data modeling verification, the anatomical conditions of the tooth and periodontium reflect the patient’s actual situation. Although we categorized the typical anatomical factors of these teeth, multiple factors can still simultaneously affect the results, causing slight deviations. Our findings indicate that these deviations are minimal (less than 2.46°). The teeth in all 31 cases were safely extracted without root fractures, suggesting that the OTA range could bring meaningful clinical guidance.

The success of the torsion method on single-rooted teeth prompted interest in its application to multiple-rooted teeth. We found that the torsion method produced significant stress concentrations in the root bifurcation region during loading, under different anatomical conditions (Supplementary Figures S8–S12). Anatomical studies have revealed the presence of multiple ridges, peaks, and pits in the root furcation region, resulting in a complex interplay between convex and concave features. (Svärdström and Wennström, 1988; Brand et al., 2023). Stress concentrations in these weak regions increase the risk of root fracture. Therefore, caution is needed when applying forces to multiple-rooted teeth with either method.

In tooth extraction under clinical settings, torsion force is often applied alongside traction force, allowing tooth dislocation without tearing all the PDL by twisting. In our study, the loading process involved only the torsion force, with complete PDL tearing as the criterion for judgment. Therefore, the obtained OTA resulted in a larger OTA than typically required in clinical, which is the maximum rotational range of the tooth. Because of the pioneering nature of this study, direct comparison with the previous reports is not possible. We believe that these results underscore the value of the torsion method, which provides clear guidance for its wider adoption in clinical practice. However, this study has limitations. We did not analyze the combined effects of multiple factors on the torsion angle, and more complex clinical scenarios need further exploration, there may be other influential anatomical variations not considered. Accurately quantifying the torsion angle using a specific tool or method in clinical practice remains a challenge. The clinical application for the torsion method can be further refined by more extensive clinical studies. Future research efforts should focus on expanding clinical validation and developing precise, minimally invasive, and quantifiable extraction tools or digital devices to meet different clinical situations.

## Conclusion

In this study, we performed biomechanical analysis to replicate PDL tearing by using the torsion method. We have expanded the application of the torsion method: single-rooted teeth with root width-to-thickness ratios of ≥0.42 and apical curvatures of ≤30°are suitable for extraction using the torsion method. Due to significant stress concentration at the root bifurcation of multi-rooted teeth during torsion, using a single force for extraction is not recommended. Furthermore, we elucidated varying patterns in torsion angles across various teeth and periodontal anatomical structures that the decrease in PDL area necessitated a larger angle for complete tearing. This study confirms the viability of the torsion method for minimally invasive tooth extraction, laying the theoretical foundation for its clinical application.

## Data Availability

The original contributions presented in the study are included in the article/Supplementary Material, further inquiries can be directed to the corresponding authors.
