# Supplementary material for: Clinical insights into tooth extraction via torsion method: a biomechanical analysis of the tooth-periodontal ligament complex
Source: Front Bioeng Biotechnol. 2024 Oct 10;12:1479751. doi: 10.3389/fbioe.2024.1479751 (PMC11500037; doi:10.3389/fbioe.2024.1479751)
Supplement: Supplementary file 1 [file Image1.PDF]

Supplementary files:

Clinical insights into tooth extraction via torsion method: a biomechanical analysis of the tooth-periodontal ligament complex

Jiawei Xing[1], Guangzeng Zhang[2], Mingliang Sun[2], Hao Pan[2], Congdi Zhang[2], Yao Liu[1], Kehan Li[1], Ze He[1], Kailiang Zhang[2], Jizeng Wang[3], En Luo[1*], Baoping Zhang[2,4*]

[1] State Key Laboratory of Oral Diseases & National Center for Stomatology & National Clinical Research Center for Oral Diseases, West China Hospital of Stomatology, Sichuan University, Chengdu 610041, Sichuan, China

[2] Department (Hospital) of Stomatology, Lanzhou University, Lanzhou, 730000, China

[3] Key Laboratory of Mechanics on Disaster and Environment in Western China, Ministry of Education, College of Civil Engineering and Mechanics, Lanzhou University, Lanzhou, 730000, China

[4] Key Lab of Maxillofacial Reconstruction and Intelligent Manufacturing, Lanzhou, 730000, Gansu China

[*]Correspondence:
En Luo
State Key Laboratory of Oral Diseases & National Center for Stomatology & National Clinical Research Center for Oral Diseases, West China Hospital of Stomatology, Sichuan University, No. 14- Section 3-Renmin South Road, Chengdu 610041, Sichuan, PR China
Email: luoen521125@sina.com

Bao-Ping Zhang
Key Lab of Maxillofacial Reconstruction and Intelligent Manufacturing, College of Stomatology, Lanzhou University, Donggang west Road 199, Lanzhou 730000, Gansu, PR China
Email: zhangbp@lzu.edu.cn

Supplementary materials and methods

Effect of time on PDL's von Mises stress

The elastic and viscoelastic models represent distinct constitutive relationships. Although the viscoelastic model is more in line with the time-dependent properties of PDL in torsion (Lee, et al., 2018), it exhibits stress relaxation phenomena dependent on the duration of stress application (Schmidt and Lapatki, 2018). Therefore, this experiment is based on the basic model, endowed with PDL viscoelastic properties, and set different torsion times of 5s, 2.5s, 1s, 0.75s, 0.5s, and 0.25s in consideration of clinical tooth extraction experience to investigate the effect of time on von Mise stress.

Supplementary Results

The viscoelastic PDL did not exhibit stress relaxation within the required timeframe of this study (5, 2.5, 1, 0.75, 0.5, and 0.25 s; FIGURE. S13). In addition, variations in time had minimal effects on OTA values (TABLE. S5). Thus, we used the linear-elastic PDL model for subsequent analyses.

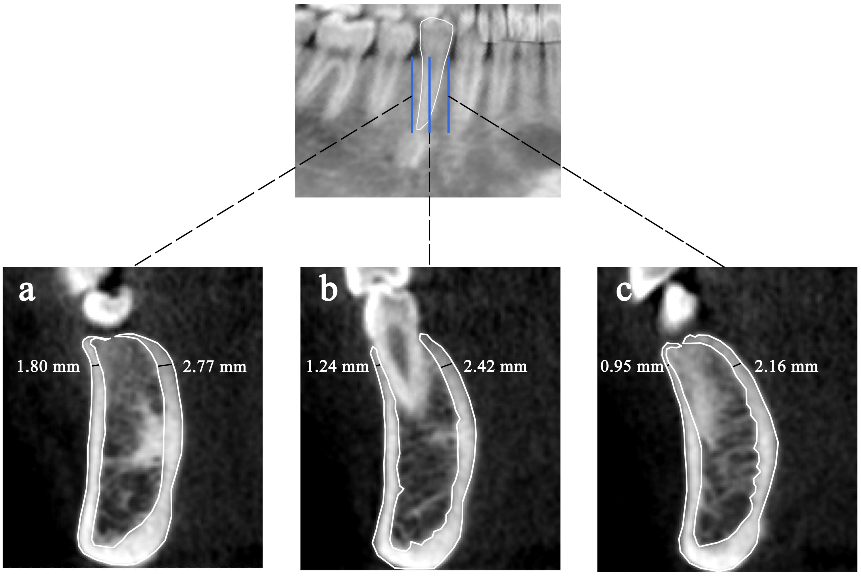

FIGURE. S1. Measuring cortical bone thickness at six areas using CBCT. In both the buccal and lingual alveolar bone regions of tooth, measurements were taken at two points each from the distal (**a**), middle (**b**), and mesial (**c**) positions along the mid-height of the tooth root. Six measurement points were considered to determine the average thickness of the cortical bone.

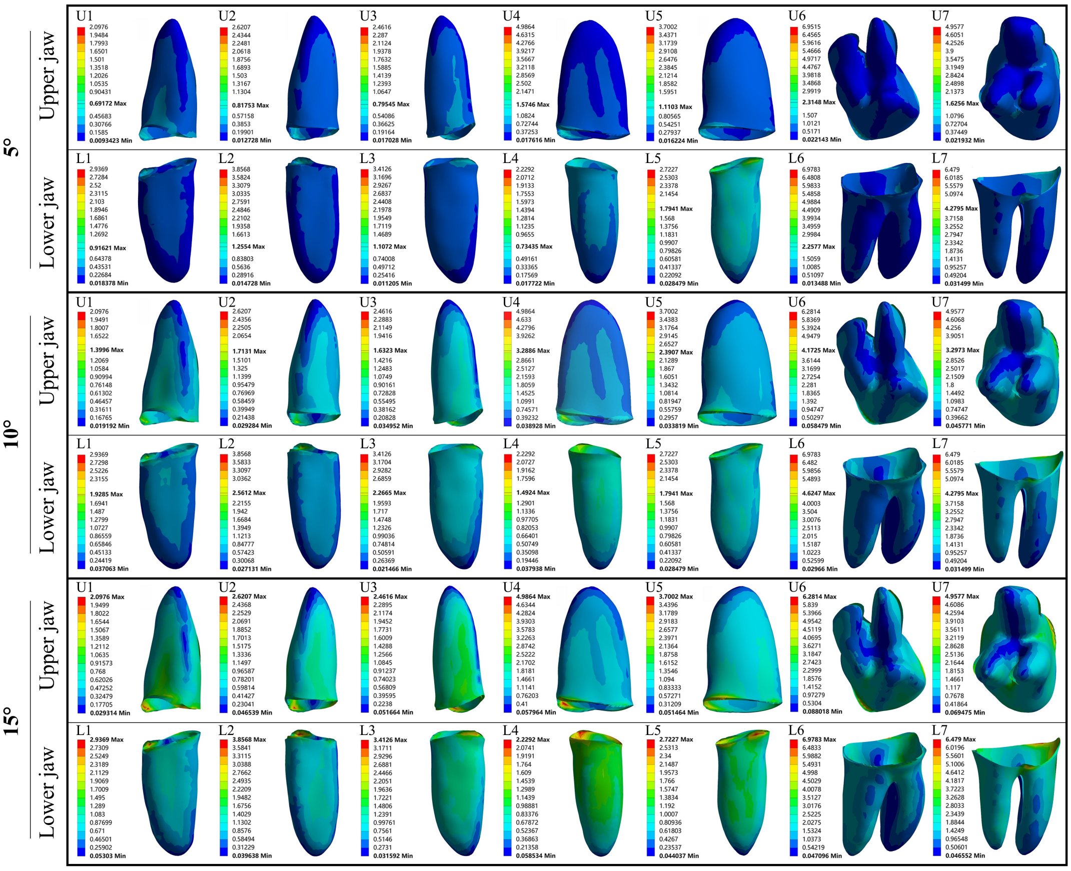

FIGURE. S2. Stress distribution of the periodontal ligament during 5°-15° torsion. U: upper jaw; L: Lower jaw.

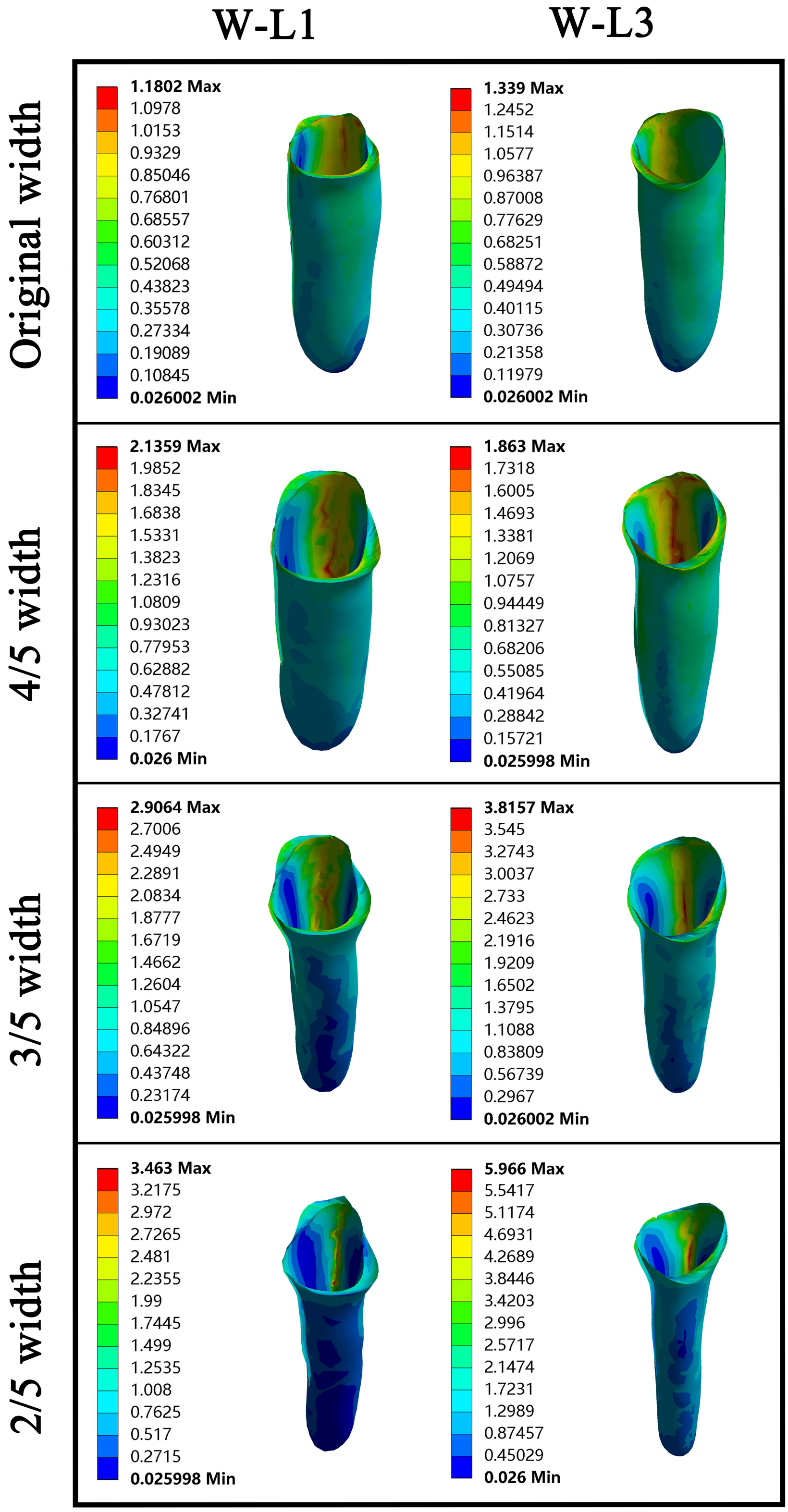

FIGURE. S3. Von Mises stress distribution in periodontal ligament of root width group at the optimal torsion angle. W-L: Root Width group-Lower jaw.

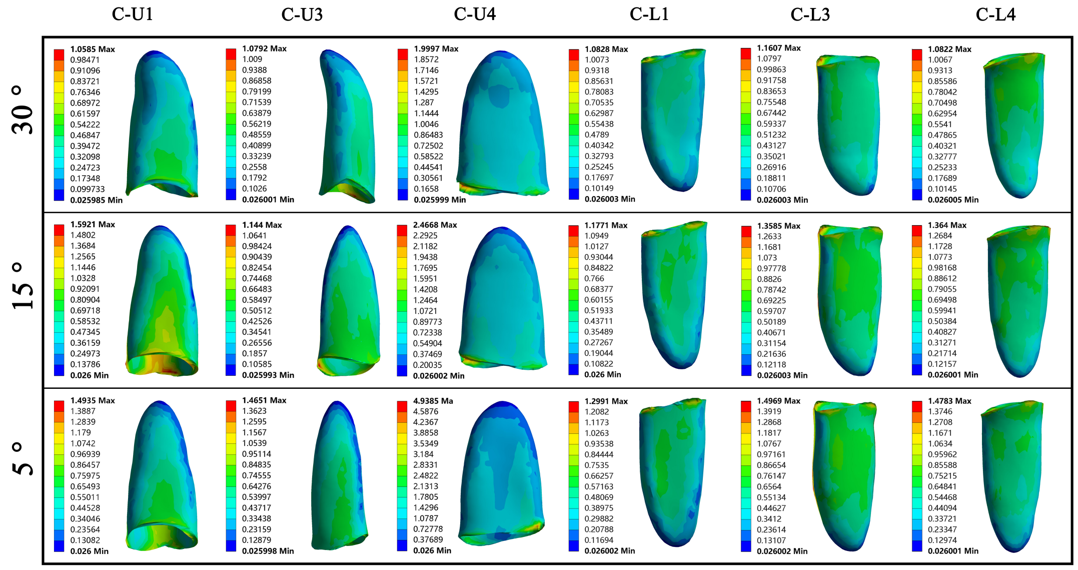

FIGURE. S4. Von Mises stress distribution in periodontal ligament of root apex curvature group at the optimal torsion angle. C-U/L: Root Apex Curvature group-Upper/Lower jaw.

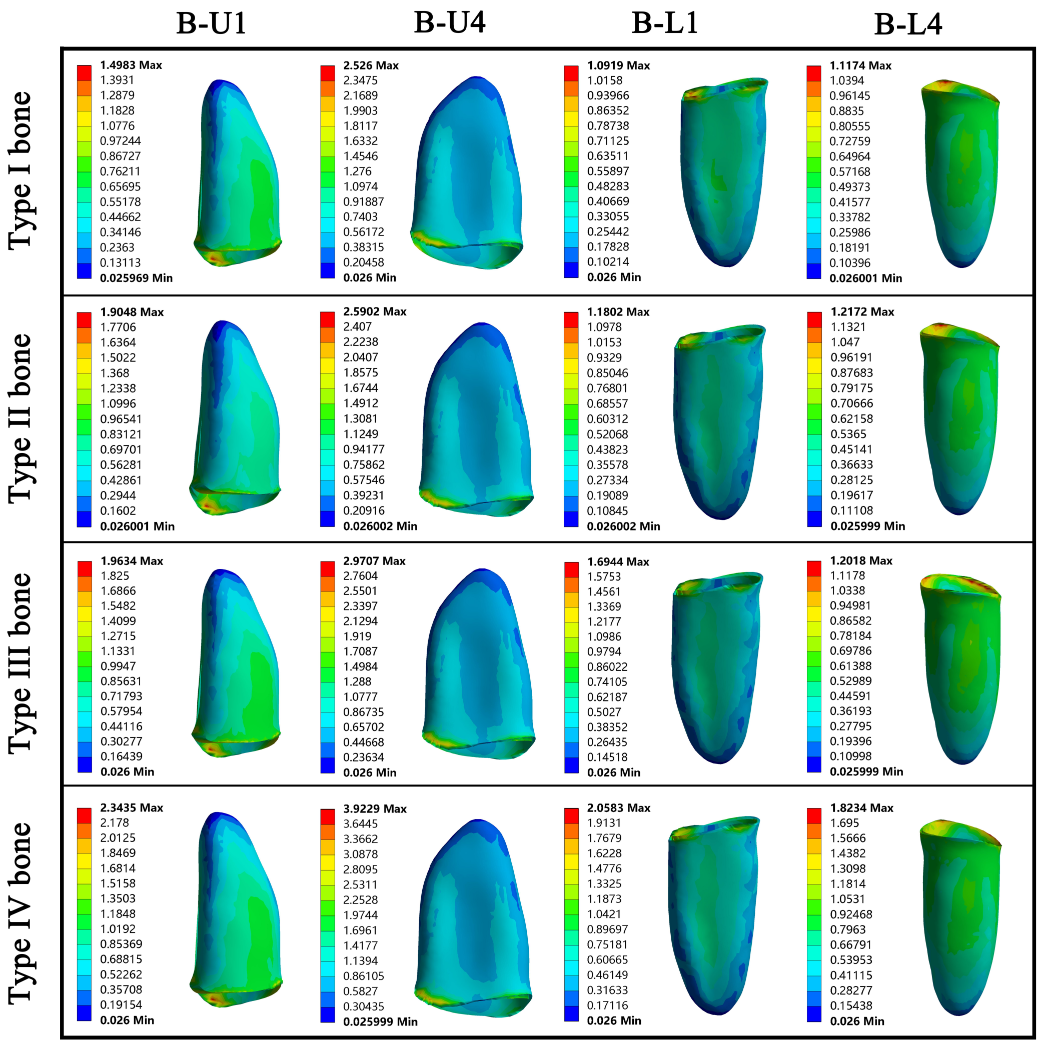

FIGURE. S5. Von Mises stress distribution in periodontal ligament of Type I to IV bone group at the optimal torsion angle. B-U/L: Type I to IV Bone group-Upper/Lower jaw.

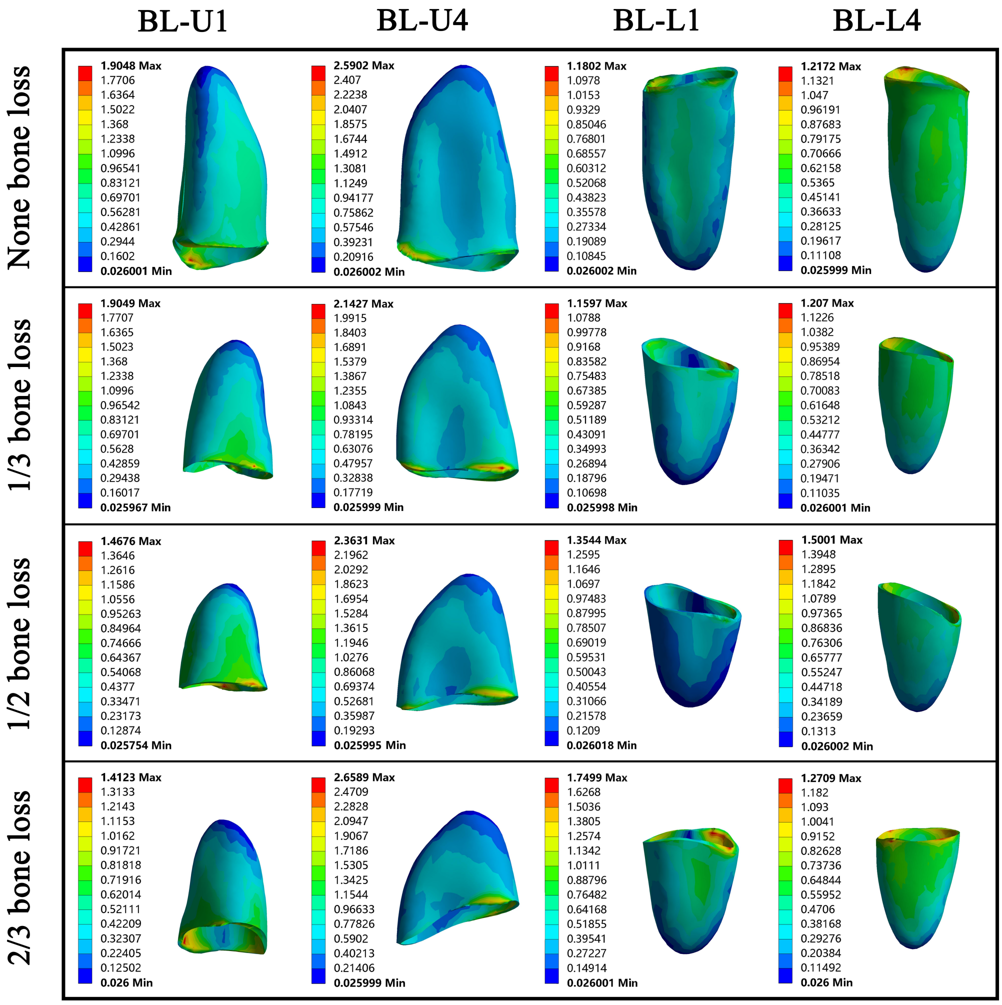

FIGURE. S6. Von Mises stress distribution in periodontal ligament of alveolar bone loss group at the optimal torsion angle. BL-U/L: Alveolar Bone Loss group-Upper/Lower jaw.

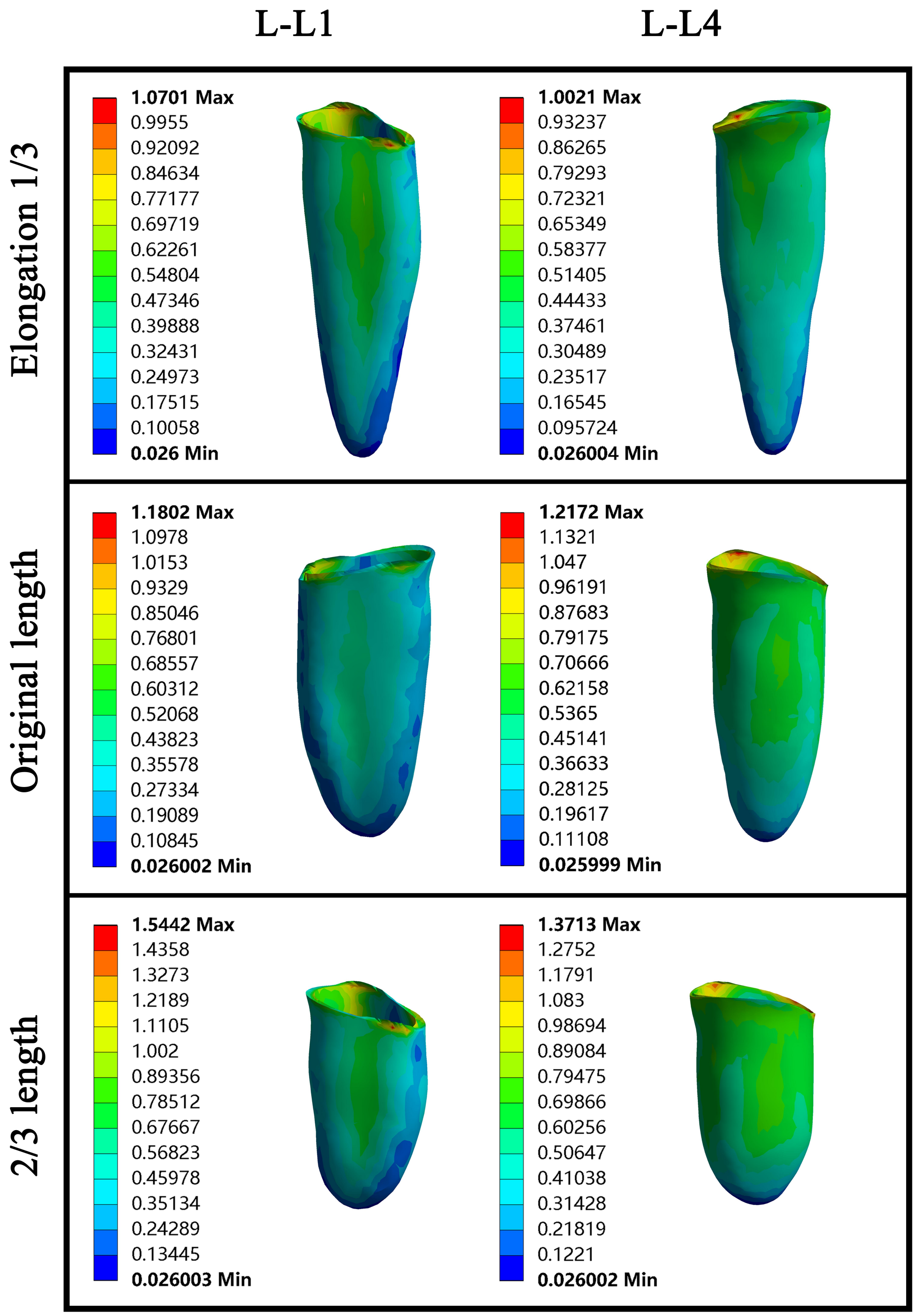

FIGURE. S7. Von Mises stress distribution in periodontal ligament of root length group at the optimal torsion angle. L-L: Root Length group-Lower jaw.

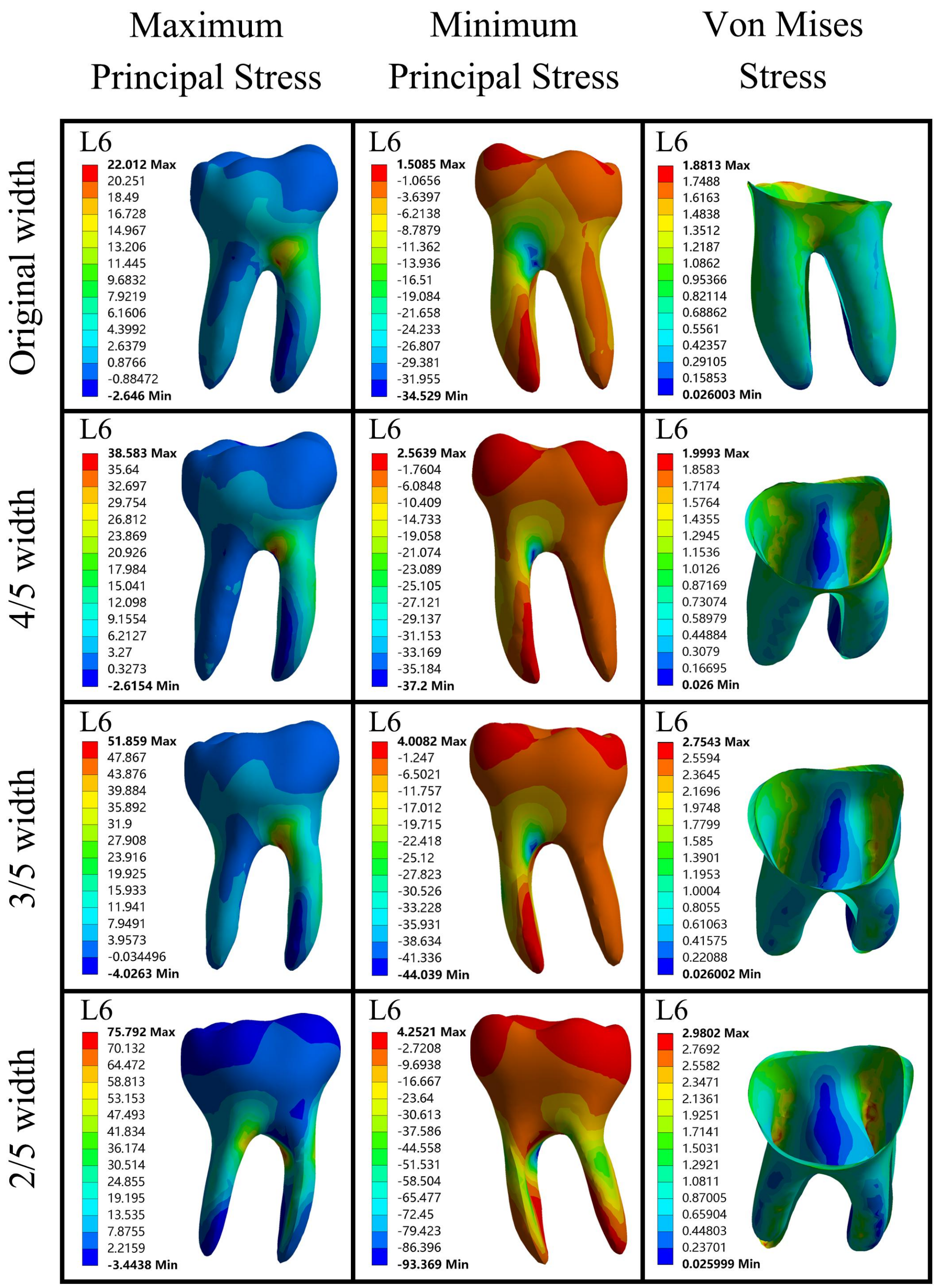

FIGURE. S8. Stress distribution of the molar in root width group during 5°-15° torsion. L: Lower jaw.

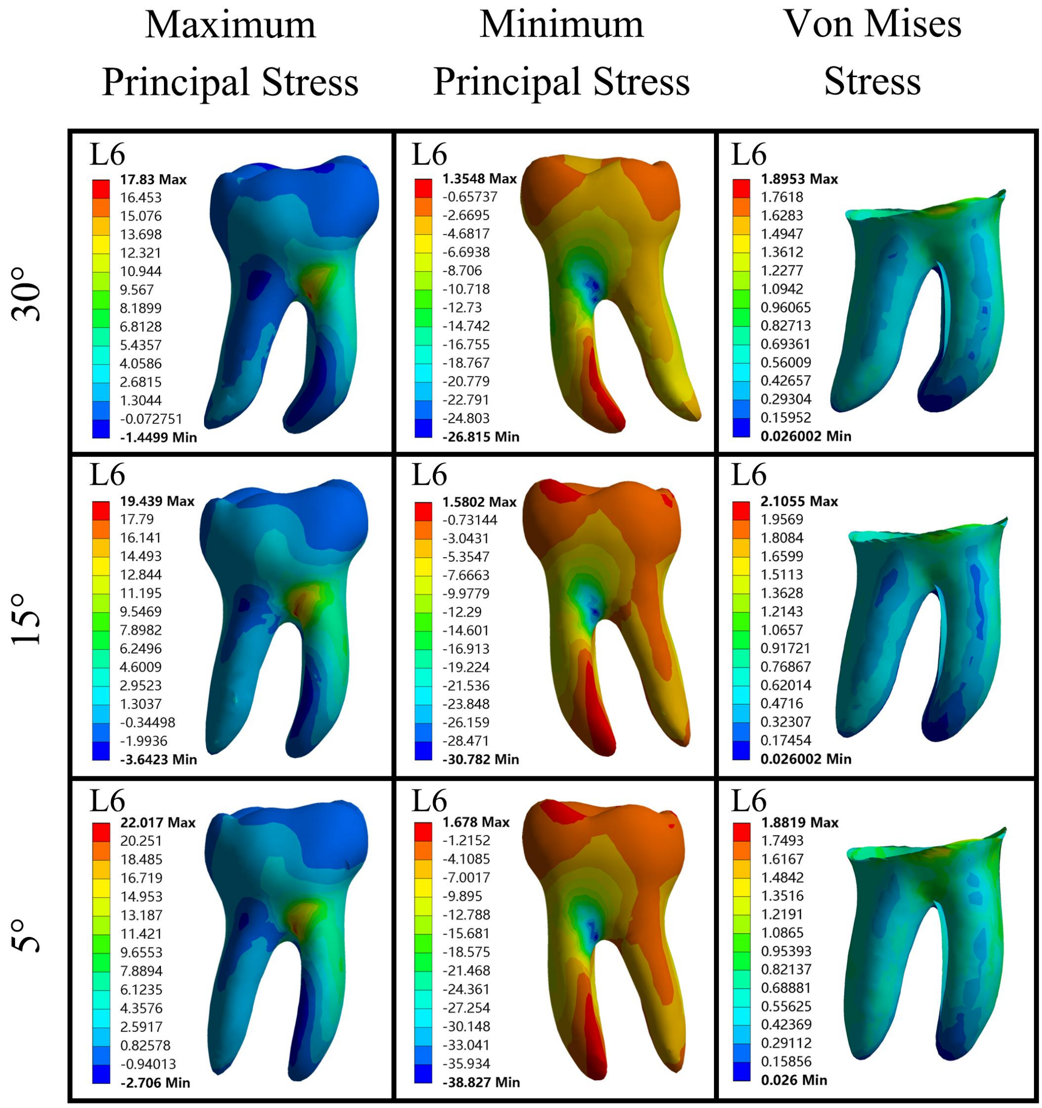

FIGURE. S9 Stress distribution of the molar in root apex curvature group during 5°-15° torsion. L: Lower jaw.

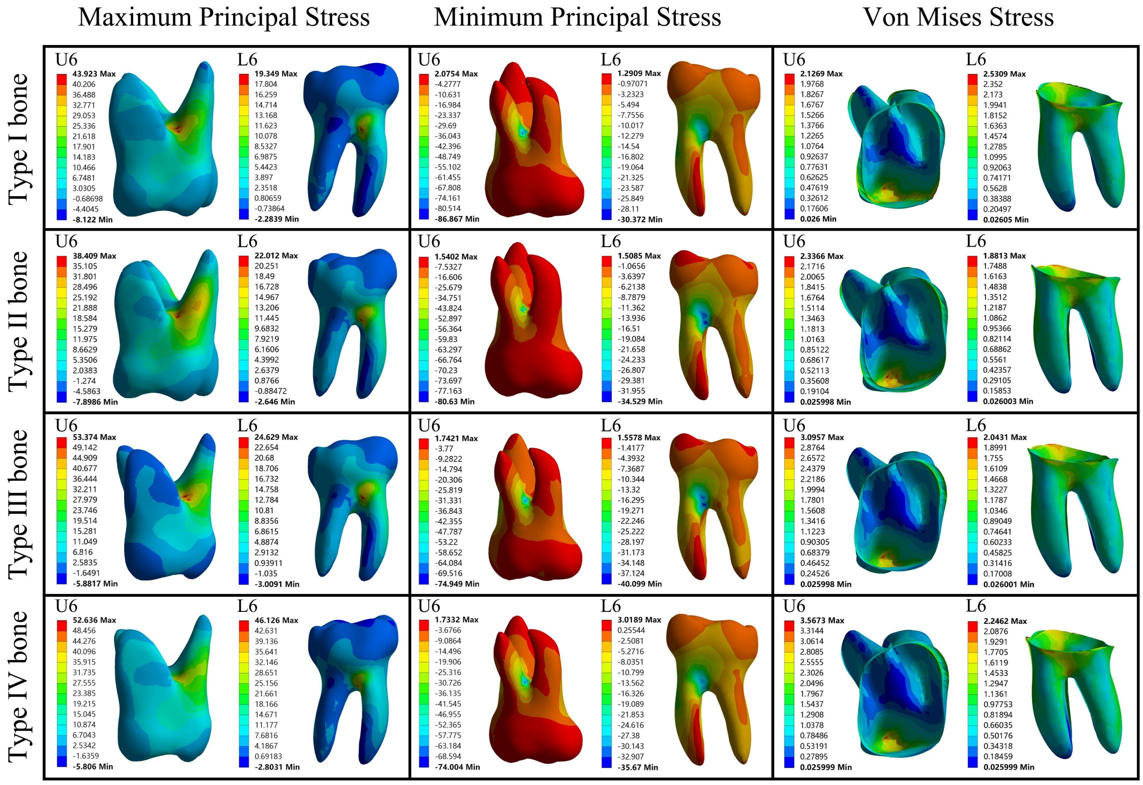

FIGURE. S10 Stress distribution of the molar in Type of bone group during 5°-15° torsion. U: upper jaw; L: Lower jaw.

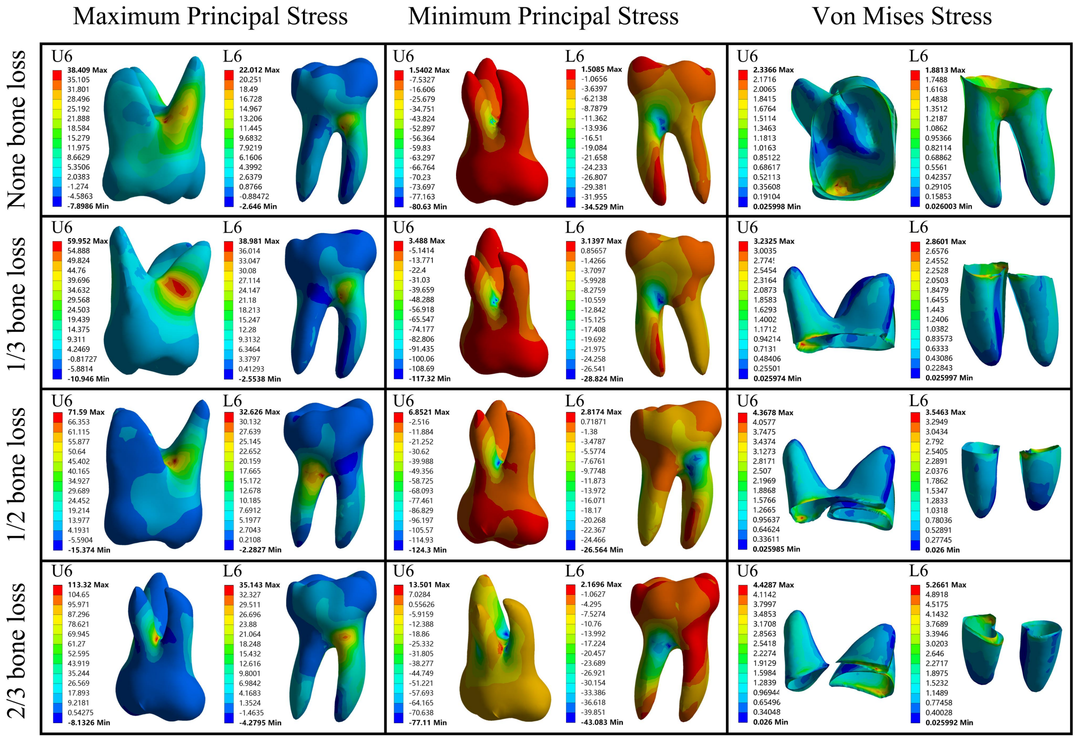

FIGURE. S11 Stress distribution of the molar in alveolar bone loss group during 5°-15° torsion. L: Lower jaw.

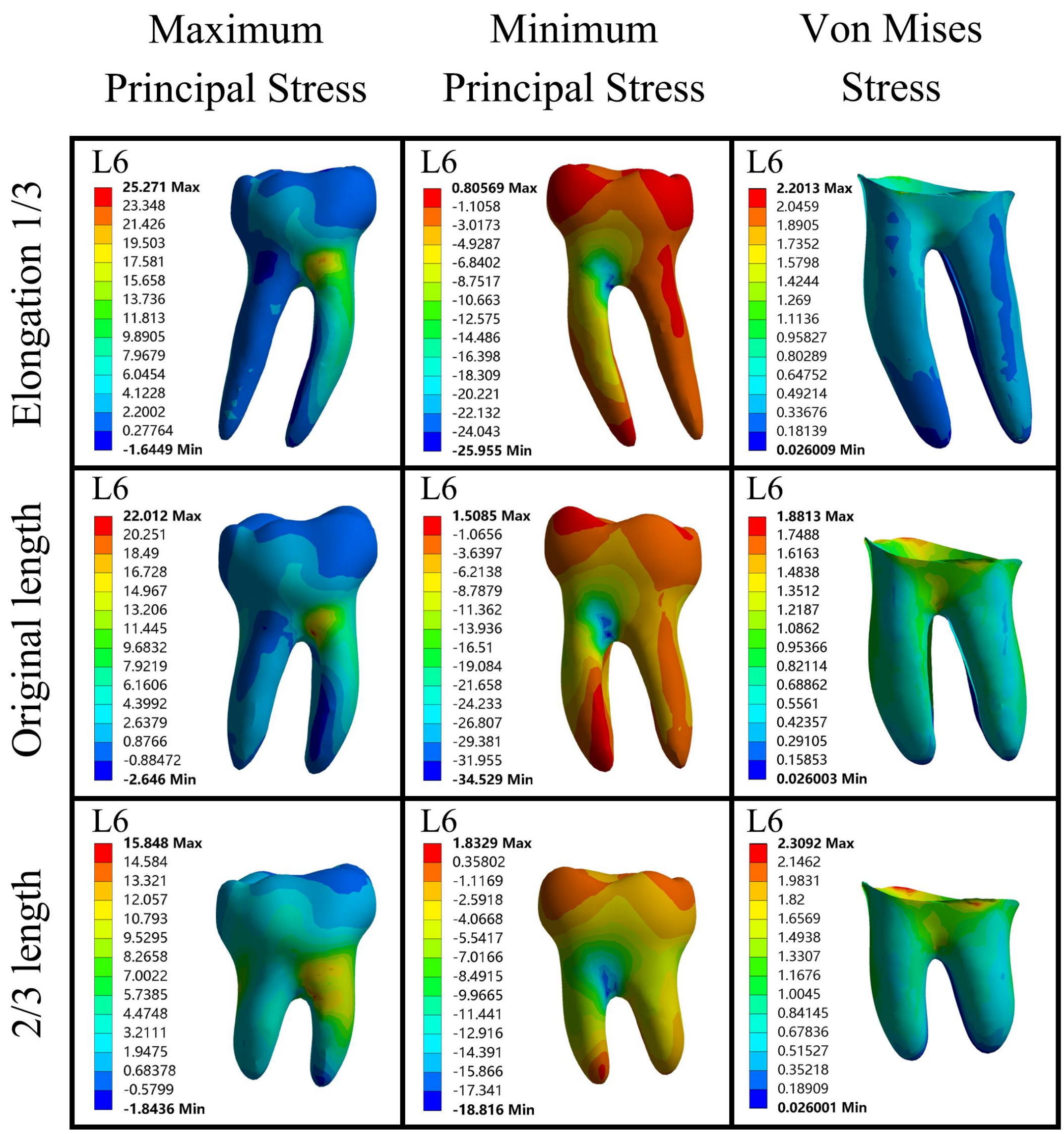

FIGURE. S12 Stress distribution of the molar in root length group during 5°-15° torsion. L: Lower jaw.

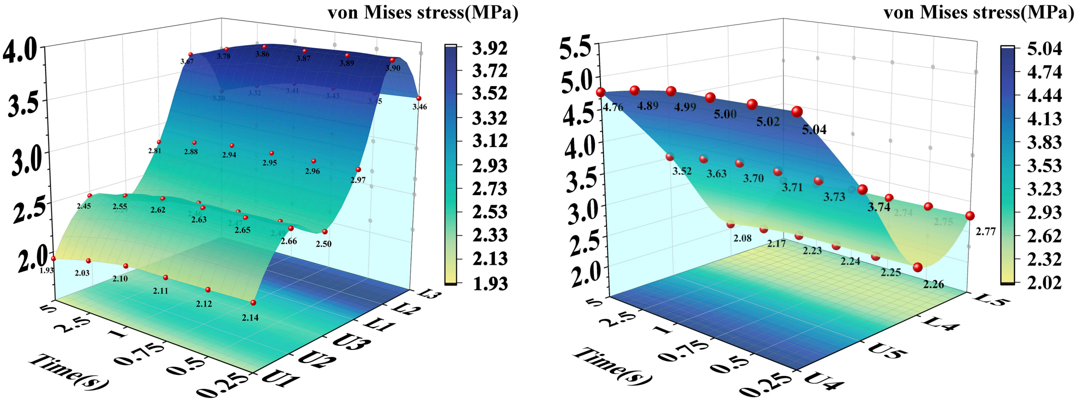

a   b

FIGURE. S13. Effect of time on maximum von Mises stress. **a** Anterior PDL group; **b** Premolar PDL group. PDL, Periodontal ligament; U, upper jaw; L, lower jaw.

TABLE S1. The nodes and elements of each model.

Model	Nodes	Elements
**Viscoelastic model group**		
U1	7911	2598
U2	5217	1742
U3	8519	2747
U4	7801	2648
U5	7226	2396
U6	17584	5879
U7	16171	5459
L1	5021	1626
L2	5414	1724
L3	8218	2723
L4	6900	2245
L5	9413	3032
L6	15364	4974
L7	15450	4974
U1 PDL	6000	2959
U2 PDL	5375	2639
U3 PDL	6935	3416
U4 PDL	8144	4020
U5 PDL	6033	2981
U6 PDL	12180	6020
U7 PDL	10226	5041
L1 PDL	3846	1878
L2 PDL	5828	2870
L3 PDL	8858	4383
L4 PDL	8470	4196
L5 PDL	9142	4520
L6 PDL	13211	6544
L7 PDL	9437	4656
U1 Maxilla	78145	49036
U2 Maxilla	84759	53578
U3 Maxilla	101750	65631
U4 Maxilla	93381	59766
U5 Maxilla	79876	50610
U6 Maxilla	94925	61115
U7 Maxilla	93506	60083
L1 Mandible	85451	53767
L2 Mandible	79206	49371
L3 Mandible	100738	64740
L4 Mandible	100914	64820

	L5 Mandible	117620	76716
	L6 Mandible	119692	78210
	L7 Mandible	106482	69186
**Type I-IV bone group**			
Type I bone	B-U1 Maxilla cortical bone	65351	43581
	B-U4 Maxilla cortical bone	111531	77011
	B-U6 Maxilla cortical bone	86724	59028
	B-L1 Mandible cortical bone	81952	54569
	B-L4 Mandible cortical bone	133490	92790
	B-L6 Mandible cortical bone	98092	66072
Type II bone	B-U1 Maxilla cortical bone	46002	27537
	B-U4 Maxilla cortical bone	70056	41827
	B-U6 Maxilla cortical bone	74207	44722
	B-L1 Mandible cortical bone	75781	44439
	B-L4 Mandible cortical bone	86309	51149
	B-L6 Mandible cortical bone	86149	51046
	B-U1 Maxilla trabecular bone	27980	18227
	B-U4 Maxilla trabecular bone	45821	29844
	B-U6 Maxilla trabecular bone	42580	27172
	B-L1 Mandible trabecular bone	41339	25746
	B-L4 Mandible trabecular bone	56975	36635
	B-L6 Mandible trabecular bone	50934	32240
Type III bone	B-U1 Maxilla cortical bone	36572	19580
	B-U4 Maxilla cortical bone	50494	26628
	B-U6 Maxilla cortical bone	53227	28229
	B-L1 Mandible cortical bone	60689	31910
	B-L4 Mandible cortical bone	66091	34909
	B-L6 Mandible cortical bone	53523	28118
	B-U1 Maxilla trabecular bone	41167	27381
	B-U4 Maxilla trabecular bone	64561	43113
	B-U6 Maxilla trabecular bone	62869	41454
	B-L1 Mandible trabecular bone	58194	37394
	B-L4 Mandible trabecular bone	85149	55790
	B-L6 Mandible trabecular bone	69848	46063
Type IV bone	B-U1 Maxilla cortical bone	36777	19730
	B-U4 Maxilla cortical bone	50494	26628
	B-U6 Maxilla cortical bone	53227	28229
	B-L1 Mandible cortical bone	148568	87113
	B-L4 Mandible cortical bone	66091	34909
	B-L6 Mandible cortical bone	64742	34139
	B-U1 Maxilla Low-density trabecular bone	41167	27381
	B-U4 Maxilla Low-density	64561	43113

	trabecular bone		
	B-U6 Maxilla Low-density trabecular bone	62869	41454
	B-L1 Mandible Low-density trabecular bone	225084	156224
	B-L4 Mandible Low-density trabecular bone	85149	55790
	B-L6 Mandible Low-density trabecular bone	80121	52729
**Alveolar bone loss group**			
None bone loss	BL-U1 PDL	6744	3296
	BL-U4 PDL	7444	3650
	BL-U6 PDL	7490	3688
	BL-L1 PDL	4465	2182
	BL-L4 PDL	8612	4248
	BL-L6 PDL	12158	6020
1/3 bone loss	BL-U1 PDL	4303	1939
	BL-U4 PDL	5703	2792
	BL-U6 PDL	9305	4576
	BL-L1 PDL	2685	1291
	BL-L4 PDL	3816	1882
	BL-L6 PDL	8190	4030
1/2 bone loss	BL-U1 PDL	3065	1469
	BL-U4 PDL	3122	1499
	BL-U6 PDL	5961	2824
	BL-L1 PDL	2220	1064
	BL-L4 PDL	3668	1767
	BL-L6 PDL	5580	2695
2/3 bone loss	BL-U1 PDL	2945	1399
	BL-U4 PDL	2560	1290
	BL-U6 PDL	7942	3782
	BL-L1 PDL	1943	912
	BL-L4 PDL	3811	1861
	BL-L6 PDL	5605	2712
**Root apex curvature group**			
5°	C-U1	6863	2208
	C-U3	11086	3635
	C-U4	11996	3978
	C-U1 PDL	5970	2914
	C-U3 PDL	6940	3407
	C-U4 PDL	8092	3959
	C-L1	6775	2258
	C-L3	11359	3830

	C-L4	13044	4436
	C-L6	20703	6937
	C-L1 PDL	4996	2455
	C-L3 PDL	9042	4456
	C-L4 PDL	9427	4630
	C-L6 PDL	12137	5973
15°	C-U1	12433	4086
	C-U3	10811	3631
	C-U4	7373	2325
	C-U1 PDL	6479	3165
	C-U3 PDL	7448	3640
	C-U4 PDL	6871	3365
	C-L1	8859	3061
	C-L3	12784	8442
	C-L4	11638	3885
	C-L6	19559	6489
	C-L1 PDL	5426	2653
	C-L3 PDL	9921	4898
	C-L4 PDL	9633	4737
	C-L6 PDL	11545	5688
30°	C-U1	7380	2362
	C-U3	10059	3370
	C-U4	11973	3826
	C-U1 PDL	5826	2871
	C-U3 PDL	7478	3672
	C-U4 PDL	6301	3096
	C-L1	9555	3235
	C-L3	9689	3302
	C-L4	11254	3795
	C-L6	16980	5693
	C-L1 PDL	5339	2617
	C-L3 PDL	9205	4545
	C-L4 PDL	9054	4478
	C-L6 PDL	12202	5984
**Root length group**			
Full length	L-L1	5714	1845
	L-L4	7049	2298
	L-L6	15065	4943
	L-L1 PDL	4465	2182
	L-L4 PDL	8612	4248
	L-L6 PDL	12158	6020
Elongation 1/3	L-L1	4835	1511
	L-L4	13581	8906

	L-L6	18707	6273
	L-L1 PDL	5499	2715
	L-L4 PDL	8434	4176
	L-L6 PDL	15600	7705
2/3 length	L-L1	3531	1186
	L-L4	6839	2213
	L-L6	13882	4585
	L-L1 PDL	3071	1492
	L-L4 PDL	6129	3007
	L-L6 PDL	9027	4409
**Root width group**			
Full width	L-L1	5714	1845
	L-L3	8366	2768
	L-L6	15065	4943
	L-L1 PDL	4465	2182
	L-L3 PDL	9983	4935
	L-L6 PDL	12158	6020
4/5 width	L-L1	5839	2028
	L-L3	7155	2343
	L-L6	17459	5709
	L-L1 PDL	4045	1961
	L-L3 PDL	8687	4247
	L-L6 PDL	12735	6273
3/5 width	L-L1	6228	2009
	L-L3	22549	7825
	L-L6	13120	4280
	L-L1 PDL	4969	2415
	L-L3 PDL	10012	4907
	L-L6 PDL	12579	6177
2/5 width	L-L1	5074	1690
	L-L3	8328	2821
	L-L6	15212	5002
	L-L1 PDL	4407	2133
	L-L3 PDL	8224	4028
	L-L6 PDL	12924	6369

Note: PDL: Periodontal Ligament; U: Upper jaw; L: Lower jaw; B-U/L: Type I to IV Bone group-Upper/Lower jaw; BL-U/L: Alveolar Bone Loss group-Upper/Lower jaw; C-U/L: Root Apex Curvature group-Upper/Lower jaw; L-L: Root Length group-Lower jaw; W-L: Root Width group-Lower jaw

.

TABLE. S2. The number of nodes and elements per clinical validation model.

Tooth ID		Model	Nodes	Elements
**Type I to IV bone group**				
T1	Type III bone	B-U1 Maxilla cortical bone	25433	13654
		B-U1 Maxilla trabecular bone	28784	18994
T2	Type III bone	B-U1 Maxilla cortical bone	20832	11090
		B-U1 Maxilla trabecular bone	29206	19708
T3	Type III bone	B-U1 Maxilla cortical bone	25483	13027
		B-U1 Maxilla trabecular bone	50804	34586
T4	Type III bone	B-U1 Maxilla cortical bone	32611	17315
		B-U1 Maxilla trabecular bone	51732	34719
T5	Type II bone	B-L1 Mandible cortical bone	27903	15959
		B-L1 Mandible trabecular bone	20860	13349
T6	Type II bone	B-L1 Mandible cortical bone	30959	18437
		B-L1 Mandible trabecular bone	22459	14624
T7	Type II bone	B-L4 Mandible cortical bone	38022	23495
		B-L4 Mandible trabecular bone	21153	13639
**Alveolar bone loss group**				
T8	Bone loss 38%	BL-U1 PDL	5182	2526
T9	Bone loss 23%	BL-L1 PDL	2834	1373
T10	Bone loss 21%	BL-L1 PDL	2793	1354
T11	Bone loss 39%	BL-L1 PDL	3079	1505
T12	Bone loss 30%	BL-L4 PDL	6290	3100
T13	Bone loss 27%	BL-L4 PDL	5349	2609
T14	Bone loss 30%	BL-L4 PDL	5733	2814
**Root width group**				
T15	72%	W-L1	9377	3158
		W-L1 PDL	5435	2642
T16	72%	W-L1	10245	3488
		W-L1 PDL	4703	2301
T17	76%	W-L1	6381	2154
		W-L1 PDL	3893	1867
T18	88%	W-L3	16085	5376
		W-L3 PDL	8098	3954
T19	85%	W-L3	11244	3714
		W-L3 PDL	5575	2711
**Root length group**				
T20	102%	L-L1	7074	2302
		L-L1 PDL	4635	2250
T21	75%	L-L4	11618	3674
		L-L4 PDL	5924	2890
T22	104%	L-L4	9544	2964

		L-L4 PDL	6965	3436
T23	115%	L-L4	23110	7166
		L-L4 PDL	7167	3532
T24	106%	L-L4	10916	3636
		L-L4 PDL	7560	3711
**Root apex curvature group**				
T25	11°	C-L3	10026	3326
		C-L3 PDL	7743	3816
T26	16°	C-L3	13086	4278
		C-L3 PDL	5348	2624
T27	15°	C-L4	8812	2775
		C-L4 PDL	5174	2521
T28	22°	C-L4	18352	5653
		C-L4 PDL	6364	3127
T29	16°	C-L4	11670	3806
		C-L4 PDL	7607	3727
T30	26°	C-L4	7387	2376
		C-L4 PDL	6175	3011
T31	14°	C-L4	10373	3337
		C-L4 PDL	7555	3716

Note: T: Tooth; PDL: Periodontal Ligament; U: Upper jaw; L: Lower jaw; B-U/L: Type I to IV Bone group-Upper/Lower jaw; BL-U/L: Alveolar Bone Loss group-Upper/Lower jaw; C-U/L: Root Apex Curvature group-Upper/Lower jaw; L-L: Root Length group-Lower jaw; W-L: Root Width group-Lower jaw.

TABLE. S3. Type I to IV bone structures. (Morgan et al., 2018; Tribst et al., 2022).

Type I to IV bone	Structure
Type I bone	Entirely homogenous cortical bone
Type II bone	1.5 mm of cortical bone surrounding trabecular bone
Type III bone	0.75 mm of cortical bone surrounding trabecular bone
Type IV bone	0.75 mm of cortical bone surrounding low-density trabecular bone

TABLE. S4. Measurement of cortical bone thickness and OTA in clinical cases.

Tooth ID	Tooth	Alveolar bone width (Mean ± SD/mm)	Group	Anatomical condition	OTA
T1	B- U1	0.72±0.22	Type I to IV bone group	Type III bone	11.21°
T2	B- U1	0.73±0.25		Type III bone	10.46°
T3	B- U1	0.61±0.26		Type III bone	10.24°
T4	B- U1	0.72±0.23		Type III bone	9.53°
T5	B- L1	1.03±0.51		Type II bone	6.42°
T6	B- L1	1.27±0.36		Type II bone	7.66°
T7	B- L4	1.64±0.35		Type II bone	7.79°
T8	BL- U1	0.97±0.45	Alveolar bone loss group	Bone loss 38%	10.04°
T9	BL- L1	1.13±0.16		Bone loss 23%	9.35°
T10	BL- L1	0.67±0.28		Bone loss 21%	6.63°
T11	BL- L1	0.88±0.28		Bone loss 39%	8.96°
T12	BL- L4	1.91±0.60		Bone loss 30%	8.24°
T13	BL- L4	1.55±0.40		Bone loss 27%	7.50°
T14	BL- L4	1.71±0.43		Bone loss 30%	7.84°
T15	W- L1	1.34±0.64	Root width group	72%	7.82°
T16	W- L1	1.08±0.46		72%	10.02°
T17	W- L1	0.87±0.36		76%	7.90°
T18	W- L3	1.24±0.40		88%	9.07°
T19	W- L3	1.72±0.35		85%	8.90°
T20	L- L1	1.03±0.22	Root length group	102%	7.83°
T21	L- L4	1.71±0.69		75%	10.05°
T22	L- L4	1.89±0.70		104%	6.77°
T23	L- L4	1.69±0.70		115%	6.52°
T24	L- L4	1.77±0.57		106%	7.02°
T25	C- L3	1.46±0.64	Root apex curvature group	11°	8.06°
T26	C- L3	1.27±0.67		16°	7.65°
T27	C- L4	1.42±0.56		15°	6.33°
T28	C- L4	2.22±0.83		22°	6.42°
T29	C- L4	1.08±0.22		16°	6.01°
T30	C- L4	1.70±0.60		26°	6.49°
T31	C- L4	1.33±0.32		14°	7.67°

Note: The tooth position, average cortical bone thickness, group categorization and OTA of 31 clinical teeth were counted. OTA: Optimal Torsion Angle T: Tooth; B-U/L: Type I to IV Bone group-Upper/Lower jaw; BL-U/L: Alveolar Bone Loss group-Upper/Lower jaw; C-U/L: Root Apex Curvature group-Upper/Lower jaw; L-L: Root Length group-Lower jaw; W-L: Root Width group-Lower jaw.

TABLE. S5. The optimal torsion angle at different torsion speeds.

Time / Tooth	5s	2.5s	1s	0.75s	0.5s	0.25s
U1	14.16°	13.97°	13.37°	13.26°	13.17°	13.08°
U2	9.00°	9.00°	9.03°	9.02°	9.03°	9.06°
U3	7.74°	7.49°	7.40°	7.44°	7.49°	7.53°
U4	7.03°	6.80°	6.71°	6.78°	6.88°	6.97°
U5	7.76°	7.78°	7.77°	7.78°	7.77°	7.74°
L1	8.25°	7.25°	6.91°	6.92°	6.95°	6.91°
L2	10.21°	10.13°	9.52°	9.30°	9.06°	8.84°
L3	11.26°	11.44°	12.24°	12.40°	12.57°	12.74°
L4	7.28°	7.15°	7.06°	7.05°	7.03°	7.02°
L5	10.24°	9.59°	9.20°	9.14°	9.09°	9.02°

Note: U: Upper jaw; L: Lower jaw.

Reference

1. Lee JS, Choi HI, Lee H, Ahn SJ, Noh G. (2018). Biomechanical effect of mandibular advancement device with different protrusion positions for treatment of obstructive sleep apnoea on tooth and facial bone: A finite element study. J. Oral. Rehabil. 45, 948-958. doi:10.1111/joor.12709
2. Schmidt F, Lapatki BG. (2019). Effect of variable periodontal ligament thickness and its non-linear material properties on the location of a tooth's centre of resistance. J. Biomech. 94, 211-218. doi:10.1016/j.jbiomech.2019.07.043.
3. Morgan EF, Unnikrisnan GU, Hussein AI. (2018). Bone Mechanical Properties in Healthy and Diseased States. Annu. Rev. Biomed. Eng. 20, 119-143. doi:10.1146/annurev-bioeng-062117-121139
4. Tribst JPM, Dal Piva AMO, Blom EJ, Kleverlaan CJ, Feilzer AJ. (2024). Dental biomechanics of root-analog implants in different bone types. J. Prosthet. Dent. 131, 905-915. doi:10.1016/j.prosdent.2022.10.005
